# Establishment of In Ovo *Salmonella* Enteritidis Infection and Synbiotic Delivery Models in Chick Embryos and Their Effects on Early Gut Health

**DOI:** 10.3390/ani16121863

**Published:** 2026-06-17

**Authors:** Riliang Liu, Jiguang Wang, Jiying Dai, Yamei Wang, Weijiang Zheng, Wen Yao

**Affiliations:** 1 College of Animal Science and Technology, Nanjing Agricultural University, Nanjing 210095, China; 2021205032@stu.njau.edu.cn (R.L.); 18707905131@163.com (J.D.); 2023205047@stu.njau.edu.cn (Y.W.); zhengweijiang@njau.edu.cn (W.Z.); 2 College of Agriculture and Forestry Science, Linyi University, Linyi 276000, China; wangjiguang20@163.com; 3 Key Lab of Animal Physiology and Biochemistry, Ministry of Agriculture and Rural Affairs of the People’s Republic of China, Nanjing Agricultural University, Nanjing 210095, China

**Keywords:** *Salmonella* Enteritidis, in ovo intervention, synbiotic, gut microbiota, chicks

## Abstract

Early microbial exposure during embryogenesis may shape post-hatch gut development in poultry, yet the effects of sublethal pathogenic exposure and in ovo synbiotics remain unclear. This model-establishment study preliminarily established in ovo *Salmonella* Enteritidis (SE) infection and synbiotic (SYN) delivery models and evaluated their effects on hatchability, cecal microbiota, intestinal morphology, epithelial turnover, and barrier function in newly hatched chicks. In one group, the air cells of specific pathogen-free White Leghorn eggs were injected with SE on embryonic day 12; in another group, a synbiotic consisting of *Lactobacillus plantarum*, *Pediococcus acidilactici*, and inulin was injected into the amniotic cavity on embryonic day 17.5. Sterile saline was injected as the vehicle-only procedural control at the corresponding time points and injection sites. Based on their impacts on hatchability, SE1-L and SYN-H were selected. SE1-L reduced cecal microbial diversity, expanded Proteobacteria and *Escherichia–Shigella*, increased ileal apoptosis and crypt depth, decreased the villus height-to-crypt depth ratio, downregulated jejunal tight-junction genes, upregulated ileal MYD88 and TNF-α, and increased plasma lipopolysaccharide and D-lactate. In contrast, SYN-H maintained hatchability, promoted early *Pediococcus* colonization, suppressed potential pathogens, increased ileal villus height and villus height-to-crypt depth ratio, enhanced proliferation, reduced apoptosis, and improved mucosal barrier-related indices. These findings provide preliminary evidence that embryonic SE infection and synbiotic delivery differentially influence early intestinal microbiota succession and gut development in newly hatched chicks.

## Simple Summary

Early life is critical for gut development and microbial colonization in chicks. However, it remains unclear how harmful or beneficial microbial exposure during embryonic development affects intestinal health after hatching. In this model-establishment study, we preliminarily established an in ovo *Salmonella* Enteritidis infection model and an in ovo synbiotic delivery model in chicks. Embryonic exposure to *Salmonella* Enteritidis disrupted cecal microbiota, increased the abundance of potential pathogenic bacteria, aggravated intestinal inflammation, and impaired intestinal barrier-related indices. In contrast, in ovo delivery of a synbiotic containing *Lactobacillus plantarum*, *Pediococcus acidilactici*, and inulin promoted early colonization by beneficial bacteria, improved ileal development, enhanced epithelial cell proliferation, and supported mucosal barrier-related parameters without reducing hatchability. These findings suggest that embryonic microbial exposure may influence early gut microbial succession and intestinal development in newly hatched chicks. The established models provide preliminary tools for studying early host–microbe interactions in chickens, although further larger-scale and longer-term validation under commercial laying-hen production conditions is needed.

## 1. Introduction

Early life represents a critical window for gastrointestinal development and immune system establishment, thereby exerting long-term effects on animal health and productivity [1]. In addition, microbial colonization of mucosal surfaces during this period plays a fundamental role in intestinal maturation and host homeostasis [2]. Increasing evidence from mammals and poultry has shown that maternal microorganisms can be vertically transmitted to the fetus and may colonize the developing gastrointestinal tract before birth or hatching, respectively [3,4,5]. However, the ecological processes by which exposure to specific microbes during embryogenesis shape post-hatch gut microbial succession and intestinal function in chicks remain poorly understood.

*Salmonella* Enteritidis (SE) is one of the most important zoonotic foodborne pathogens in intensive poultry production, causing substantial economic losses and posing a serious threat to public health through the contamination of poultry products [6,7]. Previous studies have shown that SE can infect embryonated eggs through either vertical or horizontal transmission, thereby threatening embryonic survival and chick quality [8]. Recent studies on bacterial infection in chicken embryos have mainly focused on pathogen virulence or embryo mortality [9]; thus, the sublethal consequences of embryonic SE exposure for intestinal development and gut health after hatching remain largely unclear. This issue is particularly important because embryos surviving nonlethal infection may still enter the production chain. Moreover, early-life pathogen exposure may disrupt intestinal homeostasis and trigger aberrant inflammatory responses, thereby adversely affecting the establishment of the intestinal ecosystem [10]. In contrast, beneficial early-life microbial colonization may provide a positive stimulus for gut development. Probiotic microorganisms can regulate gut microbial composition and promote immune maturation in poultry [11]. Synbiotics, which combine probiotics with prebiotic substrates, may provide additional benefits by selectively enhancing the survival and activity of beneficial strains [12].

Therefore, in the present study, poultry-derived SE strains were used to establish in ovo infection models, whereas a synbiotic composed of *Pediococcus acidilactici*, *Lactobacillus plantarum*, and inulin was used to establish an in ovo intervention model [12]. By screening treatment groups according to hatchability and subsequently evaluating newly hatched chicks, this model-establishment study aimed to establish embryonic microbial intervention models and compare the immediate post-hatch effects of sublethal SE infection and synbiotic delivery on cecal microbiota, intestinal morphology, epithelial cell turnover, mucosal barrier function, and inflammatory responses. This work may provide an experimental basis for elucidating early host–microbe interactions in poultry and developing in ovo microbial strategies to improve early intestinal health in chicks.

## 2. Materials and Methods

### 2.1. Bacterial Strains and Inoculum Preparation

All bacterial strains used in this study were isolated from poultry intestines. Two *Salmonella* Enteritidis (SE) strains were kindly provided by Professor Wei Zhang, Nanjing Agricultural University, Nanjing, China. One strain each of *Pediococcus acidilactici* (LAB69) and *Lactobacillus plantarum* (LAB72) was provided by Professor Xiaomei Bie, Nanjing Agricultural University, Nanjing, China. Inulin was supplied by Fengning Ping’an High-tech Industry Co., Ltd. (Chengde, China; purity ≥ 99%; batch no. 2022040133602).

Aliquots of each bacterial isolate were thawed before use. The SE strains were cultured in Luria–Bertani (LB) medium (Haibo, Qingdao, China; HB-LB2023) at 37 °C for 24 h using a 1% inoculation volume. LAB69 and LAB72 were cultured in de Man, Rogosa and Sharpe (MRS) medium (Haibo, Qingdao, China; HB-MRS2023) at 37 °C for 48 h. Bacterial cells were harvested by centrifugation at 6000× *g*, washed three times with sterile saline, and quantified spectrophotometrically at 625 nm using a SHIMADZU UV-2550 spectrophotometer (Shimadzu, Kyoto, Japan). For in ovo inoculation, SE suspensions were adjusted to 1 × 10^2^ CFU/egg (low dose) or 1 × 10^3^ CFU/egg (high dose), whereas LAB69 and LAB72 suspensions were adjusted to 1 × 10^3^ CFU/egg (low dose) or 1 × 10^5^ CFU/egg (high dose). Each inoculum was diluted in sterile saline, and 200 μL was administered per egg. The synbiotic (SYN) solution was prepared by mixing *P. acidilactici* and *L. plantarum* cultures with inulin (1.76 mg/200 μL). The detailed inoculation scheme is summarized in Table 1.

### 2.2. Eggs, Incubation Conditions, and In Ovo Injection

Specific pathogen-free (SPF) White Leghorn eggs were used to minimize background microbial contamination and provide a controlled baseline for establishing the in ovo microbial exposure models. A total of 200 SPF White Leghorn eggs (52.83 ± 0.75 g) from 38-week-old breeders were obtained from the Shandong Academy of Agricultural Sciences (Jinan, China). Eggs were incubated in an automatic incubator (HD-A528, Hongde Co., Ltd., Dezhou, China) under standardized conditions described previously [13]. Infertile eggs and eggs containing dead embryos identified by candling on E11 were excluded before treatment allocation. From the remaining fertilized eggs, 175 eggs were selected and randomly allocated to seven treatment groups, with 25 fertilized eggs per group. This trial was designed as an initial model-establishment and screening experiment to evaluate the feasibility and biosafety of different in ovo SE and SYN treatments. At this screening stage, hatchability was calculated at the treatment-group level and used as the primary criterion for selecting representative treatment groups for subsequent post-hatch analyses.

On embryonic day 12 (E12), when the chorioallantoic membrane is highly vascularized, SE inocula were injected into the air cell to mimic embryonic exposure to pathogenic bacteria during incubation [14]. At the same time point, 200 μL of sterile saline was injected into the air cells of the CON and SYN group eggs. On embryonic day 17.5 (E17.5), when embryos begin swallowing amniotic fluid, SYN inocula were injected into the amniotic cavity to enable direct exposure of the gastrointestinal tract to the synbiotic preparation [15]. At this time point, 200 μL of sterile saline was injected into the amniotic cavity of eggs in the CON and SE groups. Thus, all groups underwent the same two-step in ovo injection procedure at the corresponding time points and injection sites, and the CON group served as a vehicle-only procedural control for both SE and SYN treatments. A separate non-injected negative control group was not included because previous work from our group showed no significant differences between non-injected eggs and saline-injected controls under comparable in ovo injection conditions [13]. Before each injection, the blunt end of each egg was disinfected with 75% ethanol, and a small hole was drilled at the corresponding injection site using a sterile punch. A sterile 1 mL syringe was used to inject the corresponding solution into either the air cell or amniotic cavity. The hole was immediately sealed with sterile tape, and the eggs were returned to the incubator. All treatment and control eggs underwent the same disinfection, puncture, injection, sealing, and incubation procedures, and all injections at each time point were completed within 1.5 h.

On the day of hatching, hatchability was recorded for each treatment group, and chick body weight was measured. Embryos that died before hatching and unhatched eggs were included in hatchability calculations but were not used for post-hatch sampling. Only clinically normal hatched chicks were eligible for downstream sampling. Six clinically normal chicks were then randomly selected from each group and euthanized by cervical dislocation; then, blood and intestinal tissue samples were taken under aseptic conditions. For downstream microbial, molecular, biochemical, and histological analyses, each chick was considered an independent biological replicate. Based on hatchability, the low-dose SE strain 1 group (SE1-L) and the high-dose synbiotic group (SYN-H) were selected as the representative models for subsequent analyses.

To further evaluate the reproducibility of the hatchability pattern observed in the model-establishment experiment, an independent larger-scale validation experiment was subsequently conducted, using the same incubation and injection procedures. Briefly, 750 eggs were initially incubated, and after removing infertile eggs and eggs containing dead embryos, 720 fertilized eggs were allocated to three treatment groups, with 240 eggs per group. Each group contained six replicate trays, with 40 eggs per replicate tray. In this validation experiment, hatchability was calculated for each replicate tray, and the results are presented in Supplementary Figure S1.

### 2.3. DNA Extraction, 16S rRNA Gene Sequencing, and Bioinformatics Analysis

Genomic DNA was extracted from cecal contents using the QIAamp FAST DNA Stool Mini Kit (QIAGEN, Hilden, Germany; 51604) according to the manufacturer’s instructions. DNA concentration and purity were determined using a NanoDrop 2000 spectrophotometer (Thermo Scientific, Wilmington, DE, USA), and DNA integrity was verified by 1% agarose gel electrophoresis. The V3–V4 region of the bacterial 16S rRNA gene was amplified using primers 341F (5′-CCTACGGGNGGCWGCAG-3′) and 806R (5′-GGACTACHVGGGTATCTAAT-3′). PCR amplification consisted of initial denaturation at 95 °C for 2 min, followed by 27 cycles of 95 °C for 30 s, 55 °C for 30 s, and 72 °C for 60 s, with a final extension at 72 °C for 5 min.

PCR products were purified by gel extraction and quantified using the QuantiFluor™ system (Promega, Madison, WI, USA; E2671). Equimolar amounts of purified amplicons were pooled and sequenced on an Illumina MiSeq platform (2 × 250 bp) by Ling’en Biotechnology Co., Ltd. (Shanghai, China). Raw sequencing data were deposited in the Sequence Read Archive (http://www.ncbi.nlm.nih.gov/sra/, accessed on 28 February 2025 (Accession Number PRJNA1229833). Amplicon sequence variants (ASVs) were generated following the QIIME2 Vsearch and DADA2 pipeline. A phylogenetic tree based on 16S rDNA sequences was constructed using MEGA version 5.0 with reference sequences retrieved from the NCBI database. Alpha diversity indices, including ACE richness and Shannon diversity, were calculated in QIIME2. Differences in beta diversity were assessed by ANOSIM and visualized using non-metric multidimensional scaling (NMDS) based on UniFrac distances. Venn diagrams were generated using the online Jvenn tool (http://jvenn.toulouse.inra.fr/app/index.html, accessed on 14 June 2026) [16]. Microbial phenotypes were predicted using BugBase (https://bugbase.cs.umn.edu/) [17], and functional profiles were inferred using PICRUSt2 against the Kyoto Encyclopedia of Genes and Genomes (KEGG) database.

### 2.4. Intestinal Histomorphology

Segments of the jejunum and ileum were fixed in 4% paraformaldehyde for 24 h, dehydrated in graded ethanol, embedded in paraffin, and sectioned longitudinally at 5 μm. Sections were stained with hematoxylin and eosin (H&E) for histomorphological evaluation, and additional sections were stained with Alcian blue/periodic acid–Schiff (AB/PAS) to quantify goblet cells. Images were captured using an Olympus microscope (Olympus Optical Co., Ltd., Beijing, China). For morphometric analysis, six samples per group were randomly selected (*n* = 6), from which two sections were prepared, and five well-oriented villi and crypts were measured on each section. The villus height (VH), crypt depth (CD), and villus height-to-crypt depth ratio (VCR) were determined using Image-Pro Plus 6.0 software (Media Cybernetics, Silver Spring, MD, USA).

### 2.5. Immunohistochemistry for Proliferating Cell Nuclear Antigen (PCNA)

Paraffin sections were deparaffinized in xylene, rehydrated through graded ethanol, and subjected to heat-induced antigen retrieval in 10 mM citrate buffer (pH 6.0) at 95 °C for 20 min. After cooling to room temperature, sections were permeabilized with 0.3% Triton X-100 in phosphate-buffered saline (PBS) for 10 min, and endogenous peroxidase activity was blocked with 3% hydrogen peroxide for 10 min. Non-specific binding was blocked by incubation with 5% bovine serum albumin (BSA) for 2 h at room temperature. Sections were then incubated overnight at 4 °C with anti-PCNA antibody (1:10,000; ab29, Abcam, Cambridge, UK). PCNA immunoreactivity was detected using a mouse IgG-SABC kit (Boster Biological Technology, Wuhan, China), followed by visualization with 3,3′-diaminobenzidine and hematoxylin counterstaining. Negative controls were prepared by omitting the primary antibody. Images were acquired using an Olympus microscope (Olympus, Tokyo, Japan; BX63), and the proportion of PCNA-positive cells in five randomly selected intact villi and crypts was quantified using Image-Pro Plus 6.0.

### 2.6. Terminal Deoxynucleotidyl Transferase-Mediated Deoxyuridine Triphosphate Nick-End Labeling (TUNEL) Assay

Apoptosis of jejunal and ileal epithelial cells was assessed using a TUNEL assay kit (Servicebio, Wuhan, China; GDP1402). Paraffin sections were deparaffinized, rehydrated, and digested with proteinase K (20 μg/mL). DNA strand breaks were labeled using terminal deoxynucleotidyl transferase together with the BrightRed labeling mix under dark conditions, and cell nuclei were counterstained with DAPI. Fluorescence images were collected using an LSM 900 confocal laser scanning microscope (ZEISS, Jena, Germany). The apoptotic index was calculated as the ratio of TUNEL-positive cells to total cells using Image-Pro Plus 6.0 software.

### 2.7. Immunofluorescence Staining for Mucin 2 (Muc2)

Paraffin-embedded jejunal and ileal tissues were sectioned at 3 μm, deparaffinized, and rehydrated as described above. Antigen retrieval was performed in sodium citrate buffer (pH 8.0). After washing with PBS, autofluorescence was quenched, and sections were blocked with 5% BSA for 2 h at room temperature. Sections were incubated overnight at 4 °C with rabbit anti-Muc2 antibody (1:100; Santa Cruz Biotechnology, Dallas, TX, USA; SC-7314). After PBS washing, sections were incubated with the secondary antibody for 50 min at room temperature in the dark. Nuclei were counterstained with DAPI, and sections were mounted with antifade mounting medium. Images were obtained using an inverted fluorescence microscope (DM16000B, Leica Microsystems, Wetzlar, Germany).

### 2.8. Gene Expression Analysis by Quantitative Real-Time PCR

Total RNA was reverse-transcribed into cDNA using the SMART First Strand cDNA Synthesis Kit (EURx, Gdańsk, Poland). Then, quantitative real-time PCR (qPCR) was performed on a LightCycler 480 system (Roche Diagnostics, Basel, Switzerland), with each sample analyzed in duplicate. Each 12.5 μL reaction contained 6.25 μL SYBR Green I dye (EURx), 1 μmol/L each of forward and reverse primers, and 140 ng cDNA. The amplification protocol consisted of initial denaturation at 95 °C for 15 min, followed by 40 cycles of 95 °C for 15 s, 58 °C for 20 s, and 72 °C for 20 s, followed by melting curve analysis. Relative mRNA expression was calculated using the 2^−ΔΔCt^ method.

Proliferation- and apoptosis-related genes (*PCNA*, *Ki67*, BCL-2-associated X protein (*Bax*); cysteine-aspartic acid protease-3 (*Caspase-3*); B-cell lymphoma-2 (*Bcl-2*)); the major mucin gene (*Muc2*); intestinal barrier-related genes (occludin (*OCLD*), claudin-1 (*CLDN-1*), claudin-2 (*CLDN-2*), claudin-3 (*CLDN-3*), zonula occludens-1 (*ZO-1*), and zonula occludens-2 (*ZO-2*)); and immune-related genes (Toll-like receptor 4 (*TLR4*), myeloid differentiation primary response gene 88 (*MYD88*), nuclear factor kappa-B (*NF-κB*), and tumor necrosis factor-α (*TNF-α*)) were targeted. *β-actin* was used as the reference gene. Primer sequences are listed in Supplementary Table S1.

### 2.9. Plasma Biomarkers of Intestinal Barrier Damage

Plasma diamine oxidase (DAO) activity and D-lactate concentration were measured using commercial kits (Nanjing Jiancheng Bioengineering Institute, Nanjing, China; DAO kit: A088-1-1; D-lactate kit: A019-3-1). Plasma lipopolysaccharide (LPS) concentration was determined using a limulus amebocyte lysate kit (Chinese Horseshoe Crab Reagent Manufactory, Xiamen, China; RFC96T) by a chromogenic endpoint assay with a detection limit of 0.01 EU/mL.

### 2.10. Statistical Analysis

Statistical analyses were performed using SPSS 26.0 software (IBM Corp., Armonk, NY, USA). Hatchability was calculated at the treatment-group level in the initial model-establishment experiment and at the replicate-tray level in the independent validation experiment. For downstream analyses, each chick was used as the biological replicate, and technical measurements, including qPCR duplicates and repeated histological or imaging measurements, were averaged before statistical analysis. Data normality and homogeneity of variance were evaluated using the Shapiro–Wilk and Levene tests, respectively. For normally distributed data with homogeneous variance, comparisons between two groups were analyzed using the independent-samples *t*-test. Non-normally distributed data were analyzed using the Mann–Whitney U test. Associations between differential microbial taxa and phenotypic indicators were assessed using Spearman’s correlation analysis. For microbial taxa, predicted phenotypes, KEGG pathways, and gene expression profiles, unadjusted *p* values were used for exploratory interpretation. Data are presented as means ± SEM. Differences were considered statistically significant at *p* < 0.05, whereas 0.05 ≤ *p* < 0.10 was considered to represent a tendency toward significance.

## 3. Results

### 3.1. Establishment of the In Ovo Salmonella Enteritidis Infection Model

**Figure 1:**
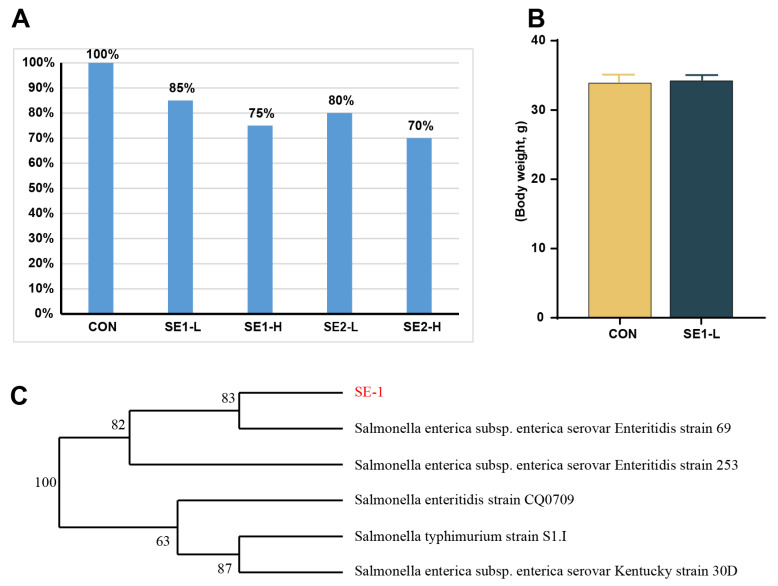
Establishment of the in ovo *Salmonella* Enteritidis infection model. (**A**) Hatchability; (**B**) hatch body weight; (**C**) 16S rRNA gene-based phylogenetic tree of the strains used in the SE model; the red label indicates SE-1. CON, saline-injected control group; SE1-L, low-dose *Salmonella* Enteritidis strain 1 group; SE1-H, high-dose *Salmonella* Enteritidis strain 1 group; SE2-L, low-dose *Salmonella* Enteritidis strain 2 group; SE2-H, high-dose *Salmonella* Enteritidis strain 2 group. For hatch body weight, data are presented as means ± SEM (*n* = 6 chicks per group).

As shown in Figure 1A, the hatchability of the CON group was 100%, whereas in ovo SE administration reduced hatchability to varying degrees, with an apparent dose-dependent pattern. To minimize the confounding effect of embryonic mortality and to further evaluate the post-hatch consequences of sublethal embryonic infection, the SE1-L group, which showed relatively high hatchability, was selected for subsequent analyses. In the independent larger-scale validation experiment, the hatchability pattern of the SE1-L group was consistent with that observed in the initial model-establishment experiment, showing a significant reduction compared with the CON group (*p* < 0.05; Figure S1). No significant difference in hatch body weight was observed between the CON and SE1-L groups (*p* > 0.05, Figure 1B). In addition, phylogenetic analysis based on 16S rRNA gene sequences showed that the isolate used in the SE1-L treatment clustered within *Salmonella enterica*, including *S.* Enteritidis reference strains, supporting its assignment at the species level rather than confirming serovar-level identity (Figure 1C).

### 3.2. Embryonic SE Infection Reshaped the Cecal Microbial Community in Newly Hatched Chicks

**Figure 2:**
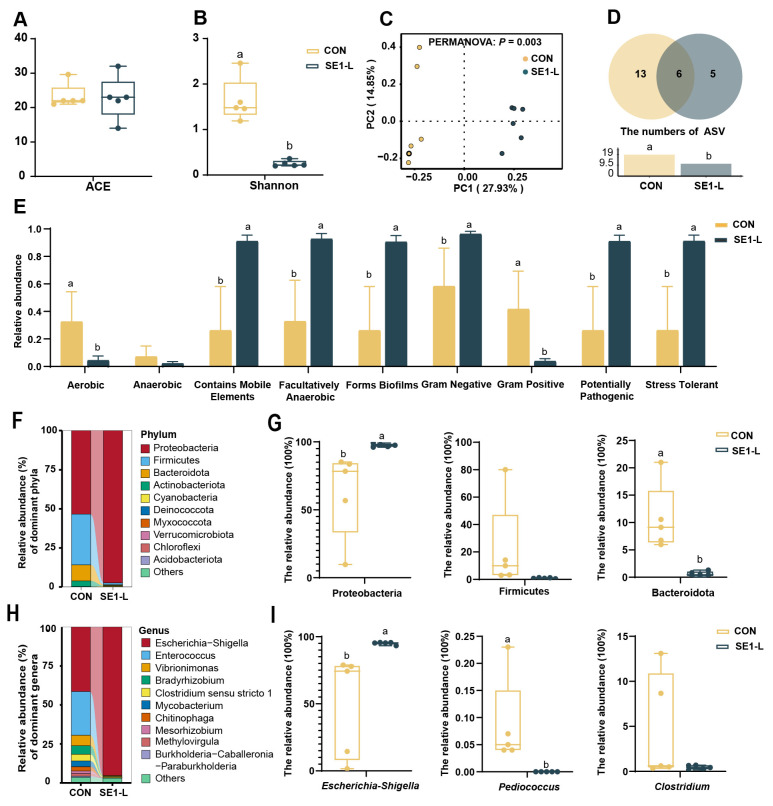
Effects of embryonic SE infection on cecal microbial composition in newly hatched chicks. (**A**) ACE index; (**B**) Shannon index; (**C**) non-metric multidimensional scaling (NMDS) plot based on UniFrac distances; (**D**) Venn diagram and ASV numbers; (**E**) microbiome phenotypes predicted by BugBase; (**F**,**G**) microbial composition and relative abundance at the phylum level; (**H**,**I**) microbial composition and relative abundance at the genus level. CON, saline-injected control group; SE1-L, low-dose *Salmonella* Enteritidis strain 1 group. Data are presented as means ± SEM (*n* = 6). Different lowercase letters indicate significant differences between groups (*p* < 0.05).

Cecal microbial profiling showed that the ACE index did not differ significantly between groups (*p* > 0.05, Figure 2A), whereas the Shannon index was significantly decreased in the SE1-L group compared with the CON group (*p* < 0.05, Figure 2B). Consistently, the Venn analysis revealed a significantly lower number of ASVs in the SE1-L group (*p* < 0.05, Figure 2D). The beta-diversity analysis further demonstrated a clear separation in microbial community structure between the CON and SE1-L groups (*p* = 0.003; Figure 2C). Using BugBase, it was predicted that the SE1-L group would show enrichment of microbiome features associated with pathogenicity, including mobile elements, facultative anaerobiosis, biofilm formation, Gram-negative phenotype, potential pathogenicity, and stress tolerance, whereas the predicted relative abundance of aerobic and Gram-positive bacteria was reduced (*p* < 0.05, Figure 2E).

At the phylum level, the SE1-L chicks were characterized by a marked expansion of Proteobacteria (97.84%), accompanied by significant reductions in Firmicutes (0.79%) and Bacteroidota (*p* < 0.05, Figure 2F,G). Additional changes were also detected in Verrucomicrobiota and Cyanobacteria (*p* < 0.05, Table S2). At the genus level, *Escherichia–Shigella* became the overwhelmingly dominant taxon in the SE1-L group, accounting for 95.51% of the total abundance, and was significantly increased relative to the CON group (*p* < 0.05, Figure 2H,I). The relative abundance of *Salmonella* was also significantly elevated in the SE1-L group (0.92%) but was undetectable in the controls (Table S2). In contrast, several genera, including *Pediococcus*, *Clostridium*, *Vibrionimonas*, and *Bradyrhizobium*, were significantly decreased in the SE1-L chicks (*p* < 0.05, Figure 2I and Table S2). The PICRUSt2-based functional prediction further suggested that SE infection was associated with alterations in a wide range of KEGG pathways (*p* < 0.05, Table S3).

### 3.3. Embryonic SE Infection Impaired Intestinal Morphology and Altered Epithelial Cell Turnover

In the jejunum, no significant differences were observed between the CON and SE1-L groups in terms of the villus height, crypt depth, or villus height-to-crypt depth ratio (VCR) (*p* > 0.05, Figure S2A,B). In contrast, in the ileum, SE1-L infection significantly increased crypt depth and significantly decreased VCR (*p* < 0.05 Figure S2C,D), while the villus height remained unchanged (*p* > 0.05, Figure S2C,D).

**Figure 3:**
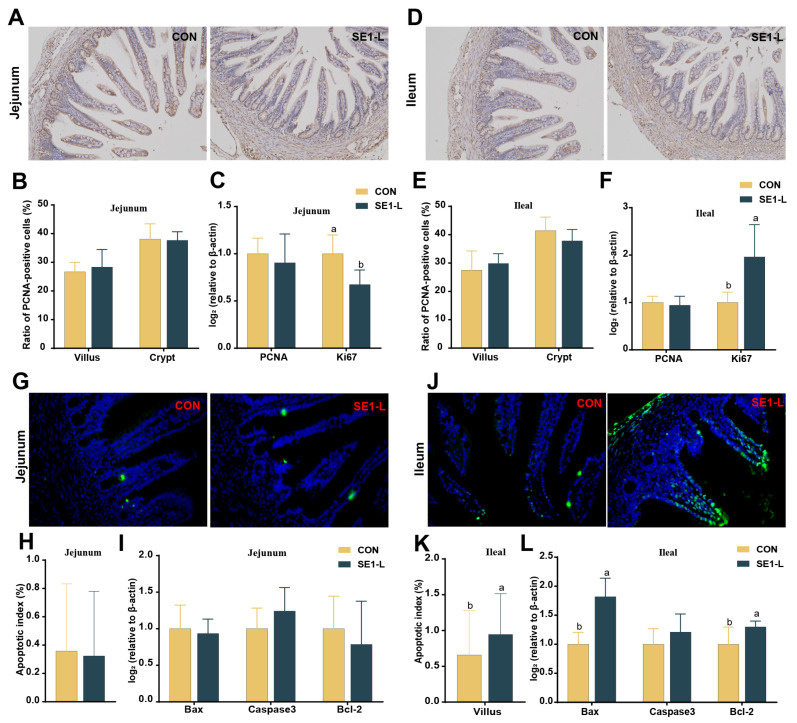
Effects of embryonic SE infection on jejunal and ileal epithelial proliferation and apoptosis in chicks. (**A**,**D**) Immunolocalization of PCNA in the jejunum and ileum; (**B**,**E**) proportions of PCNA-positive cells in villi and crypts of the jejunum and ileum; (**C**,**F**) relative mRNA expression of proliferation-related genes in the jejunum and ileum; (**G**,**J**) TUNEL staining of the jejunum and ileum; (**H**,**K**) apoptotic index of the jejunum and ileum; (**I**,**L**) relative mRNA expression of apoptosis-related genes in the jejunum and ileum. CON, saline-injected control group; SE1-L, low-dose *Salmonella* Enteritidis strain 1 group. Data are presented as means ± SEM (*n* = 6). Different lowercase letters indicate significant differences between groups (*p* < 0.05).

An analysis of epithelial proliferation showed that, in the jejunum, the proportion of PCNA-positive cells in villi and crypts, as well as PCNA mRNA abundance, did not differ significantly between the groups (*p* > 0.05, Figure 3A–C); however, jejunal *Ki67* expression was significantly lower in the SE1-L chicks than in the controls (*p* < 0.05, Figure 3C). In the ileum, neither the proportion of PCNA-positive cells nor *PCNA* mRNA expression differed significantly (*p* > 0.05, Figure 3D–F), whereas *Ki67* expression was significantly increased in the SE1-L group (*p* < 0.05, Figure 3F).

The apoptosis analysis further showed that no significant differences were detected in the jejunal apoptotic index or in the *Bax*, *Caspase-3*, and *Bcl-2* expression between the two groups (*p* > 0.05, Figure 3G–I). In the ileum, however, the villus apoptotic index was significantly increased in the SE1-L group, and both *Bax* and *Bcl-2* were significantly upregulated (*p* < 0.05, Figure 3J–L).

### 3.4. Embryonic SE Infection Disrupted Intestinal Immune and Barrier Function

Goblet cell staining showed no significant differences in the number of AB-positive cells in jejunal crypts or PAS-positive cells along the jejunal villi between the CON and SE1-L groups (*p* > 0.05, Figure S3A–C). In the ileum, the number of AB-positive goblet cells in crypts was significantly lower in the SE1-L group (*p* < 0.05, Figure S3E), whereas the number of PAS-positive cells on villi was not significantly affected (*p* > 0.05, Figure S3D–F).

**Figure 4:**
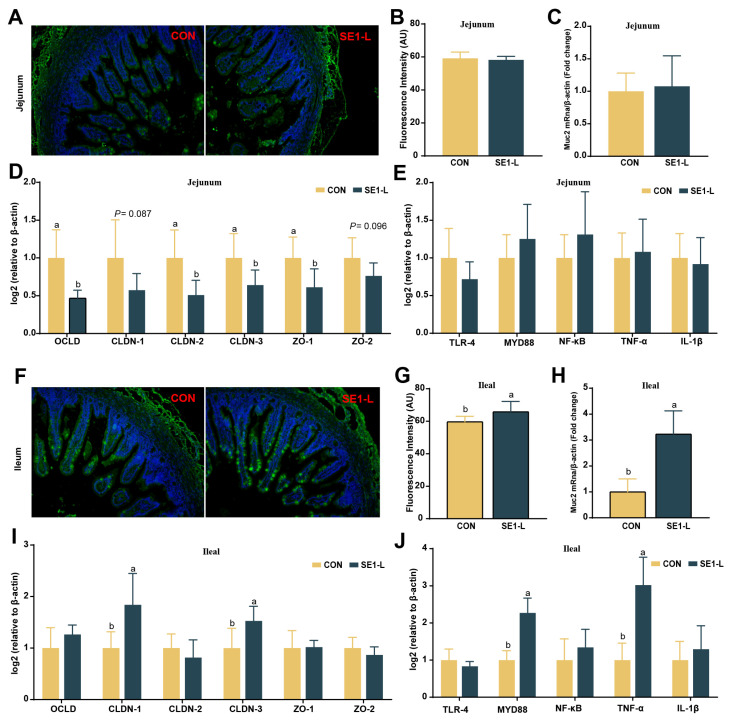
Effects of embryonic SE infection on immune- and barrier-related parameters in the jejunum and ileum of chicks. (**A**) Muc2 immunofluorescence staining in the jejunum; (**B**) quantification of jejunal Muc2 fluorescence intensity; (**C**) relative jejunal *Muc2* mRNA expression; (**D**) relative mRNA expression of barrier-related genes in the jejunum; (**E**) relative mRNA expression of inflammation-related genes in the jejunum; (**F**) Muc2 immunofluorescence staining in the ileum; (**G**) quantification of ileal Muc2 fluorescence intensity; (**H**) relative ileal *Muc2* mRNA expression; (**I**) relative mRNA expression of barrier-related genes in the ileum; (**J**) relative mRNA expression of inflammation-related genes in the ileum. CON, saline-injected control group; SE1-L, low-dose *Salmonella* Enteritidis strain 1 group. Data are presented as means ± SEM (*n* = 6). Different lowercase letters indicate significant differences between groups (*p* < 0.05).

In the jejunum, SE1-L infection did not significantly affect Muc2 fluorescence intensity or *Muc2* mRNA expression (*p* > 0.05, Figure 4A–C). However, the expression of several barrier-related genes, including OCLD, CLDN-2, CLDN-3, and ZO-1, was significantly downregulated (*p* < 0.05, Figure 4D), while no significant changes were detected in inflammatory gene expression (*p* > 0.05, Figure 4E). In the ileum, both Muc2 fluorescence intensity and Muc2 mRNA abundance were significantly increased in the SE1-L group (*p* < 0.05, Figure 4F–H). In parallel, CLDN-1 and CLDN-3 expression levels were significantly upregulated, and the inflammatory mediators MYD88 and TNF-α were also significantly increased (*p* < 0.05, Figure 4I,J).

Systemic indicators of barrier injury further supported these findings (Table S4). Plasma LPS and D-lactate concentrations were significantly elevated in the SE1-L chicks compared with the controls (*p* < 0.05), whereas DAO activity was not significantly different between the groups (*p* > 0.05).

### 3.5. Establishment of the In Ovo Synbiotic Intervention Model

**Figure 5:**
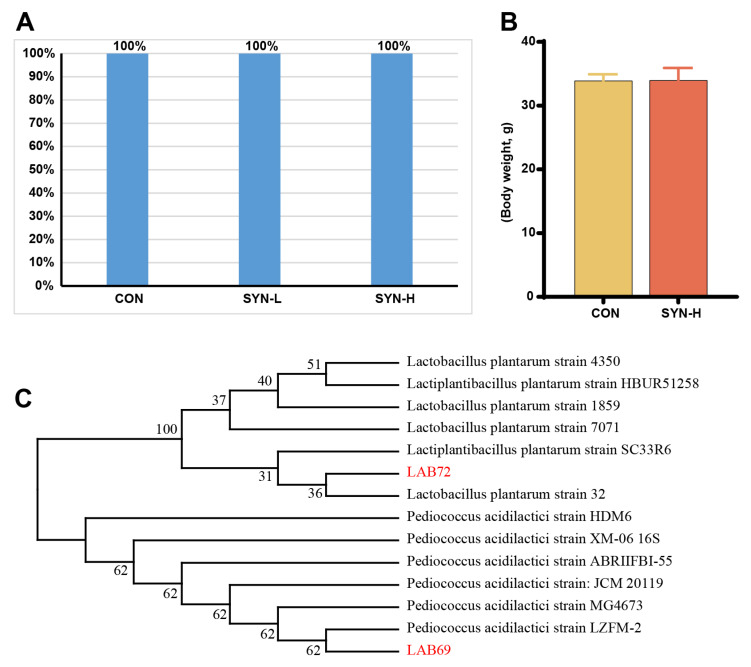
Establishment of the in ovo synbiotic intervention model. (**A**) Hatchability; (**B**) hatch body weight; (**C**) 16S rRNA gene-based phylogenetic tree of the strains used in the synbiotic model; the red labels indicate LAB72 and LAB69. CON, saline-injected control group; SYN-L, low-dose synbiotic group; SYN-H, high-dose synbiotic group. For hatch body weight, data are presented as means ± SEM (*n* = 6 chicks per group).

As shown in Figure 5A, neither SYN-L nor SYN-H significantly affected hatchability compared with the CON group. Based on these results, the SYN-H group was selected for subsequent analyses. In the independent larger-scale validation experiment, the hatchability pattern of the SYN-H group was also consistent with that observed in the initial model-establishment experiment, with hatchability remaining comparable to that of the CON group (*p* > 0.05; Figure S1). Hatch body weight also did not differ significantly between the CON and SYN-H groups (*p* > 0.05, Figure 5B). A phylogenetic analysis based on 16S rRNA gene sequences further confirmed that LAB69 and LAB72 were *Pediococcus acidilactici* and *Lactobacillus plantarum*, respectively (Figure 5C).

### 3.6. Embryonic Synbiotic Administration Modulated the Cecal Microbiota of Newly Hatched Chicks

**Figure 6:**
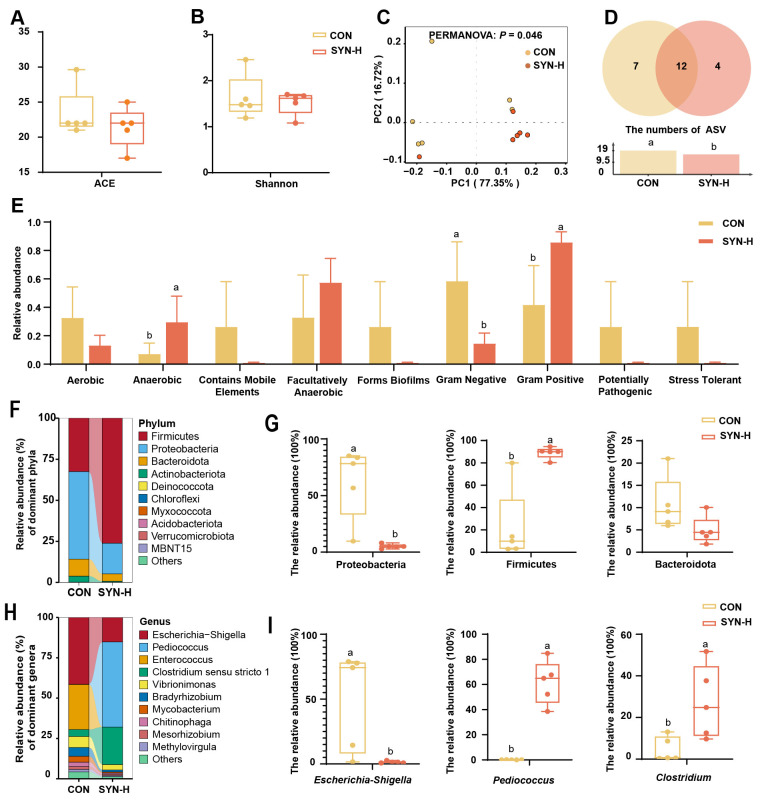
Effects of embryonic synbiotic intervention on cecal microbial composition in newly hatched chicks. (**A**) ACE index; (**B**) Shannon index; (**C**) principal coordinate analysis (PCoA) plot; (**D**) Venn diagram and ASV numbers; (**E**) microbiome phenotypes predicted by BugBase; (**F**,**G**) microbial composition and relative abundance at the phylum level; (**H**,**I**) microbial composition and relative abundance at the genus level. CON, saline-injected control group; SYN-H, high-dose synbiotic group. Data are presented as means ± SEM (*n* = 6). Different lowercase letters indicate significant differences between groups (*p* < 0.05).

No significant differences were detected between the CON and SYN-H groups in either the ACE or Shannon index (*p* > 0.05, Figure 6A,B). Nevertheless, the number of ASVs was significantly reduced in the SYN-H group (*p* < 0.05, Figure 6D), and the beta-diversity analysis showed a significant separation in microbial composition between the groups (*p* = 0.046; Figure 6C). BugBase predicted that the SYN-H microbiota was characterized by higher relative abundances of anaerobic and Gram-positive bacteria and a lower predicted relative abundance of Gram-negative bacteria (*p* < 0.05, Figure 6E).

At the phylum level, the relative abundance of Firmicutes was significantly increased (*p* < 0.05), whereas Proteobacteria was significantly decreased in the SYN-H group (*p* < 0.05); the abundance of Bacteroidota remained unchanged (*p* > 0.05, Figure 6F,G). At the genus level, Pediococcus was a dominant taxon in the SYN-H chicks and was almost undetectable in the controls. In addition, Clostridium sensu stricto 1 was significantly enriched, whereas Escherichia–Shigella was significantly reduced (*p* < 0.05, Figure 6H,I). Other genera, including Rhodanobacter and Burkholderia–Caballeronia–Paraburkholderia, were also significantly decreased (*p* < 0.05, Table S5). PICRUSt2-based functional prediction suggested that synbiotic administration was associated with predicted alterations in KEGG pathways (*p* < 0.05, Table S6).

### 3.7. Embryonic Synbiotic Administration Improved Intestinal Morphology and Epithelial Turnover

No significant differences were observed in the jejunal villus height, crypt depth, or VCR between the CON and SYN-H groups (*p* > 0.05, Figure S4A,B). In the ileum, however, SYN-H administration significantly increased villus height and VCR (*p* < 0.05), while crypt depth remained unchanged (*p* > 0.05, Figure S4C,D).

**Figure 7:**
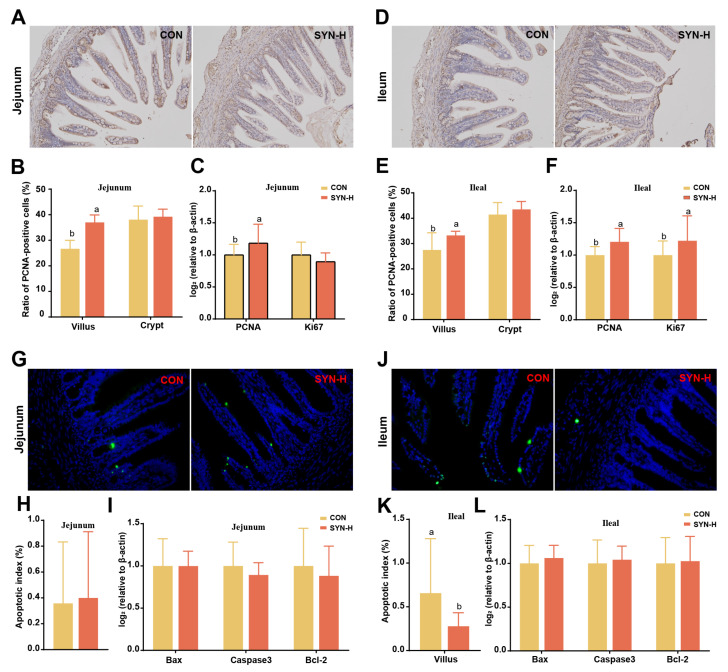
Effects of embryonic synbiotic intervention on jejunal and ileal epithelial proliferation and apoptosis in chicks. (**A**,**D**) Immunolocalization of PCNA in the jejunum and ileum; (**B**,**E**) proportions of PCNA-positive cells in villi and crypts of the jejunum and ileum; (**C**,**F**) relative mRNA expression of proliferation-related genes in the jejunum and ileum; (**G**,**J**) TUNEL staining of the jejunum and ileum; (**H**,**K**) apoptotic index of the jejunum and ileum; (**I**,**L**) relative mRNA expression of apoptosis-related genes in the jejunum and ileum. CON, saline-injected control group; SYN-H, high-dose synbiotic group. Data are presented as means ± SEM (*n* = 6). Different lowercase letters indicate significant differences between groups (*p* < 0.05).

Regarding epithelial proliferation, the proportion of PCNA-positive cells in jejunal villi was significantly higher in the SYN-H group than in the CON group (*p* < 0.05, Figure 7B), and jejunal *PCNA* mRNA expression was also significantly upregulated (*p* < 0.05, Figure 7C); by contrast, no significant differences were found in the proportion of PCNA-positive cells in jejunal crypts or in *Ki67* expression (*p* > 0.05, Figure 7B,C). In the ileum, the SYN-H treatment significantly increased the proportion of PCNA-positive cells in villi and significantly upregulated both *PCNA* and *Ki67* expression (*p* < 0.05, Figure 7D–F).

The apoptosis analysis showed that the jejunal apoptotic index and *Bax*, *Caspase-3*, and *Bcl-2* expression were not significantly altered by SYN-H treatment (*p* > 0.05, Figure 7G–I). In the ileum, however, the villus apoptotic index was significantly reduced in the SYN-H group (*p* < 0.05, Figure 7J), whereas the expression of apoptosis-related genes remained unchanged (*p* > 0.05, Figure 7L).

### 3.8. Embryonic Synbiotic Administration Maintained Intestinal Barrier Integrity While Modulating Mucosal Immunity

No significant differences were observed in the numbers of AB-positive or PAS-positive goblet cells in either the jejunum or ileum between the CON and SYN-H groups (*p* > 0.05, Figure S5A–F).

**Figure 8:**
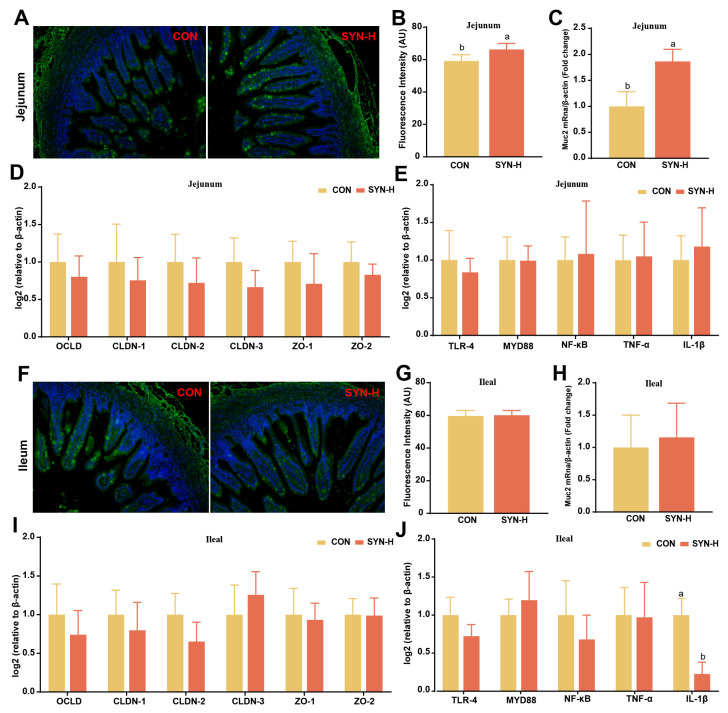
Effects of embryonic synbiotic intervention on immune and barrier functions in the jejunum and ileum of chicks. (**A**) Muc2 immunofluorescence staining in the jejunum; (**B**) quantification of jejunal Muc2 fluorescence intensity; (**C**) relative jejunal *Muc2* mRNA expression; (**D**) relative mRNA expression of barrier-related genes in the jejunum; (**E**) relative mRNA expression of inflammation-related genes in the jejunum; (**F**) Muc2 immunofluorescence staining in the ileum; (**G**) quantification of ileal Muc2 fluorescence intensity; (**H**) relative ileal *Muc2* mRNA expression; (**I**) relative mRNA expression of barrier-related genes in the ileum; (**J**) relative mRNA expression of inflammation-related genes in the ileum. CON, saline-injected control group; SYN-H, high-dose synbiotic group. Data are presented as means ± SEM (*n* = 6). Different lowercase letters indicate significant differences between groups (*p* < 0.05).

In the jejunum, SYN-H significantly increased Muc2 fluorescence intensity and Muc2 mRNA expression compared with the CON group (*p* < 0.05, Figure 8A–C). However, no significant differences were detected in the expression of barrier- or inflammation-related genes (*p* > 0.05, Figure 8D,E). In the ileum, neither Muc2 fluorescence intensity nor Muc2 mRNA expression differed significantly between groups (*p* > 0.05, Figure 8F–H). Likewise, the expression of ileal barrier-related genes was not significantly altered by SYN-H treatment (*p* > 0.05, Figure 8I). Among immune-related genes, only IL-1β was significantly decreased in the ileum of the SYN-H chicks (*p* < 0.05), whereas other inflammatory markers remained unchanged (*p* > 0.05, Figure 8J).

Consistent with these local findings, plasma concentrations of LPS and D-lactate, as well as DAO activity, were not significantly different between the CON and SYN-H groups (*p* > 0.05, Table S7).

### 3.9. Correlation Analysis Between Cecal Microbiota and Host Developmental and Immune Traits

**Figure 9:**
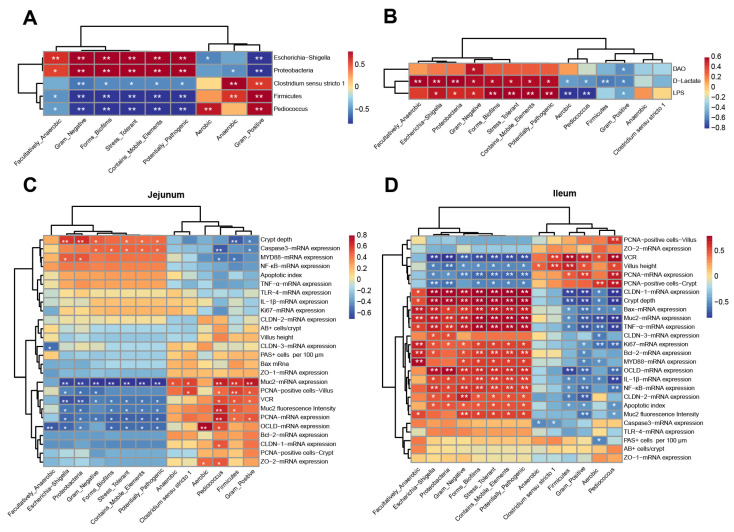
Correlation analysis between cecal microbiota and host developmental and immune parameters in chicks. (**A**) Correlation analysis between key differential microorganisms and microbiome phenotypes; (**B**) correlation analysis between key differential microorganisms, microbial phenotypes, and plasma intestinal barrier damage biomarkers; (**C**,**D**) correlation analysis between key differential microorganisms, microbial phenotypes, and developmental and immune-related parameters in the jejunum and ileum. Red indicates positive correlations, and blue indicates negative correlations (* 0.01 < *p* < 0.05; ** 0.001 < *p* < 0.01).

As shown in Figure 9A, Proteobacteria and Escherichia–Shigella were positively correlated with microbiome phenotypes associated with pathogenicity, including facultative anaerobiosis, Gram-negative phenotype, biofilm formation, stress tolerance, mobile elements, and potential pathogenicity (*p* < 0.05). In contrast, Firmicutes, Pediococcus, and Clostridium sensu stricto 1 were negatively correlated with these phenotypic traits (*p* < 0.05).

Plasma D-lactate and LPS levels were positively correlated with Proteobacteria, Escherichia–Shigella, and pathogenic microbial phenotypes, but negatively correlated with Gram-positive bacteria, Pediococcus, and Clostridium sensu stricto 1 (*p* < 0.05, Figure 9B). Moreover, in both the jejunum and ileum, Proteobacteria and Escherichia–Shigella were negatively correlated with indices associated with intestinal health, such as VCR and the expression of PCNA, *Muc2*, and *OCLD*, whereas they were positively correlated with inflammatory and apoptosis-related markers, including *Caspase-3*, *MYD88*, *TNF-α*, *IL-1β*, and *NF-κB* (*p* < 0.05, Figure 9C,D). Firmicutes, Pediococcus, and Gram-positive taxa exhibited the opposite correlation pattern (*p* < 0.05, Figure 9C,D).

## 4. Discussion

The present study established in ovo microbial intervention models and compared the early post-hatch effects of embryonic exposure to SE or a synbiotic on gut microbiota assembly, intestinal development, and barrier function in chicks. Under commercial conditions, embryos that die from infection are discarded, whereas survivors of nonlethal infection may still enter the production chain, with their subsequent health status often overlooked [9,18]. Here, different SE strains and doses reduced hatchability to different extents. Accordingly, SE1-L was selected as the representative model, indicating that a sublethal embryonic SE infection model was successfully established. Importantly, the hatchability pattern of the selected SE1-L group was further supported by an independent larger-scale validation experiment using replicate trays, in which the SE1-L group showed reduced hatchability compared with that of the CON group.

Early-life gut microbiota is a key determinant of long-term host health and productivity [19]. In this study, embryonic SE infection reduced cecal microbial diversity and ASV number and markedly shifted beta diversity, indicating disrupted microbial succession. Reduced diversity is generally considered a sign of delayed microbiota maturation and increased intestinal vulnerability [20]. BugBase predictions further suggested the enrichment of biofilm-forming, Gram-negative, and potentially pathogenic microbial phenotypes [21,22]. At the taxonomic level, the marked expansion of Proteobacteria and *Escherichia–Shigella* is particularly important, as both are recognized indicators of intestinal dysbiosis and opportunistic pathogen overgrowth [23]. Although *Salmonella* itself was enriched, its abundance remained lower than that of *Escherichia–Shigella*, suggesting that embryonic SE exposure triggered broader ecological instability rather than only increasing the inoculated pathogen. This agrees with previous findings identifying *Escherichia–Shigella* expansion as a marker of *Salmonella*-associated dysbiosis in newly hatched chicks [24]. Overall, these results suggest that embryonic SE infection disturbed early microbial succession.

Early life is also a critical period for intestinal development, and microbial exposure during this window of time can markedly influence epithelial maturation [25,26]. In the present study, SE infection increased the ileal crypt depth and reduced VCR, accompanied by enhanced epithelial apoptosis. These changes are typical of epithelial injury and abnormal tissue renewal [27]. The elevated expression of *Bax* further supports the occurrence of epithelial stress and damage, whereas the increase in ileal *Ki67* may reflect compensatory proliferation in response to epithelial loss [28]. Overall, embryonic SE exposure impaired intestinal structural development by disturbing the balance between epithelial injury and repair.

Early microbial succession is a key driver of gut-associated lymphoid tissue development and local immune homeostasis, and disruption of this process may directly impair intestinal barrier function [29]. In the present study, although SE infection reduced the number of AB-positive goblet cells in ileal crypts, both Muc2 fluorescence intensity and *Muc2* mRNA abundance were significantly increased, which may represent a compensatory mucus secretory response to pathogen challenge [30]. The concurrent upregulation of *MYD88* supports this interpretation, as MYD88-mediated signaling is known to contribute to host protection against enteric infection [31]. However, this compensation did not fully offset barrier injury. In the jejunum, the downregulation of *OCLD*, *CLDN-2*, *CLDN-3*, and *ZO-1* indicated impaired tight-junction assembly and weakened physical barrier integrity. In the ileum, the significant upregulation of *MYD88* and *TNF-α* suggested the activation of inflammatory signaling. In particular, increased *TNF-α* expression may enhance intestinal permeability through ERK- and JNK-related pathways [32,33]. Collectively, these findings indicate that embryonic SE infection impaired intestinal barrier function through coordinated disruption of tight junctions, goblet cell responses, and inflammatory activation.

In contrast, in ovo synbiotic administration provided a beneficial microbial stimulus during embryogenesis. In poultry, the early administration of probiotics or synbiotics is considered a promising strategy for modulating gut development, provided that biosafety is ensured [34]. In the present study, neither low- nor high-dose synbiotic treatment affected hatchability or hatch weight, and SYN-H was therefore selected as the representative synbiotic delivery model for subsequent analyses. Consistent with the initial model-establishment experiment, the independent larger-scale validation experiment showed that the SYN-H group exhibited a level of hatchability comparable to that of the CON group.

Unlike SE infection, synbiotic administration did not significantly alter the alpha diversity, although it clearly restructured the beta diversity. BugBase predictions suggested the enrichment of Gram-positive and anaerobic microbial phenotypes in the SYN-H group, consistent with the increased abundance of *Pediococcus* and *Clostridium sensu stricto 1*. At the community level, SYN-H increased Firmicutes and reduced Proteobacteria, a shift generally associated with microbiota maturation and improved intestinal health [35]. At the genus level, the marked increase in *Pediococcus* suggests successful early colonization by beneficial bacteria after in ovo delivery. At the same time, *Escherichia–Shigella* was significantly suppressed, possibly due to competitive exclusion by beneficial microbes through antimicrobial metabolites or preferential occupation of mucosal adhesion sites [36,37]. The predicted enrichment of pathways related to replication and repair; translation; and folding, sorting, and degradation may indicate altered microbial functional potential after synbiotic intervention. Overall, embryonic synbiotic administration was associated with a cecal microbial profile characterized by a lower predicted pathogenic potential and altered functional capacity.

At the host level, the synbiotic treatment improved intestinal development. In the ileum, SYN-H increased the villus height and VCR, enhanced epithelial proliferation, and reduced apoptosis. Specifically, the proportions of PCNA-positive cells and the expression of *PCNA* and *Ki67* were significantly increased in the jejunum and/or ileum, whereas the ileal apoptotic index was significantly reduced. Enhanced proliferation implies accelerated enterocyte turnover and may facilitate the establishment of a more functional absorptive surface during the immediate post-hatch period [38].

In addition, the synbiotic treatment improved mucosal barrier function. In the jejunum, although the goblet cell number was unchanged, SYN-H significantly increased Muc2 fluorescence intensity and *Muc2* mRNA expression. Because Muc2 is a major component of the mucus layer, this increase may strengthen the first chemical barrier against pathogen colonization [39]. Unlike the stress-associated mucus response observed after SE infection, the increase after synbiotic treatment more likely reflects developmental enhancement of the mucosal barrier [40]. In the ileum, SYN-H reduced *IL-1β* expression while maintaining stable barrier-related gene expression, and systemic barrier biomarkers remained low. These findings indicate that embryonic synbiotic delivery supported early intestinal development and barrier homeostasis by promoting mucus production and reducing local inflammatory tone.

The correlation analysis further linked microbial succession with host phenotypes. Proteobacteria and *Escherichia–Shigella* were positively associated with pathogenic phenotypes, elevated plasma LPS and D-lactate, and pro-inflammatory markers such as *MYD88* and *TNF-α*. In contrast, Firmicutes and *Pediococcus*, enriched in the SYN group, were positively associated with Gram-positive and anaerobic phenotypes and showed favorable correlations with VCR, *PCNA*, and *Muc2*. Thus, the microbial shifts induced by SE and SYN were not merely compositional but were closely related to intestinal development, barrier integrity, and immune status. Overall, the present study suggests that embryonic exposure to *Salmonella* Enteritidis or synbiotics differentially affected early intestinal microbiota assembly, intestinal development, and barrier-related indices in newly hatched chicks.

## 5. Conclusions

This study preliminarily established in ovo models of *Salmonella* Enteritidis exposure and synbiotic delivery in chicks. Under the experimental conditions described, embryonic SE exposure was associated with gut dysbiosis, epithelial injury, inflammatory activation, and impaired barrier-related indices, whereas synbiotic delivery promoted beneficial bacterial colonization and supported early intestinal development without reducing hatchability. These findings provide preliminary evidence that embryonic microbial exposure may influence early intestinal microbiota succession and gut health in newly hatched chicks. However, some microbiota-, function-, and gene-related findings were exploratory because multiple testing correction was not applied. Given the relatively small sample size for downstream analyses and the absence of long-term validation of production performance, tissue colonization, metabolomic outcomes, and multi-strain formulations, further larger-scale studies in laying-hen lines under commercial production conditions are warranted.

## Figures and Tables

**Table 1:** In ovo inoculation protocol used to establish the embryonic *Salmonella* Enteritidis infection and synbiotic intervention models.

Treatment ^1^	E12 Air-Cell Inoculum	E17.5 Amniotic Cavity Inoculum
CON	200 μL of 0.9% sterile saline	200 μL of 0.9% sterile saline
SE1-L	1 × 10^2^ CFU *S*. Enteritidis	200 μL of 0.9% sterile saline
SE1-H	1 × 10^3^ CFU *S*. Enteritidis 1	200 μL of 0.9% sterile saline
SE2-L	1 × 10^2^ CFU *S*. Enteritidis 2	200 μL of 0.9% sterile saline
SE2-H	1 × 10^3^ CFU *S*. Enteritidis 2	200 μL of 0.9% sterile saline
SYN-L	200 μL of 0.9% sterile saline	1 × 10^3^ CFU each of LAB69 and LAB72 + 1.76 mg inulin
SYN-H	200 μL of 0.9% sterile saline	1 × 10^5^ CFU each of LAB69 and LAB72 + 1.76 mg inulin

^1^ CON, saline-injected control group; SE, *Salmonella* Enteritidis; SYN, synbiotic; SE1 and SE2 indicate *Salmonella* Enteritidis strain 1 and strain 2, respectively; LAB69, *Pediococcus acidilactici*; LAB72, *Lactobacillus plantarum*; L and H indicate low and high doses, respectively. All inocula were administered in a final volume of 200 μL per egg.

## Data Availability

The raw reads were deposited into the NCBI Sequence Read Archive (SRA) database (Accession Number: PRJNA1229833).

## Supplementary Materials

The following supporting information can be downloaded at https://www.mdpi.com/article/10.3390/ani16121863/s1, Table S1. Primers used for real-time PCR analysis; Table S2. Effects of embryonic SE infection on the relative abundance of cecal microbiota at the phylum and genus levels; Table S3. Effects of embryonic SE infection on the predicted functional KEGG pathways of cecal microbiota in chicks; Table S4. Effects of embryonic SE infection on plasma biomarkers of intestinal barrier damage in chicks; Table S5. Effects of embryonic synbiotic intervention on the relative abundance of cecal microbiota at the phylum and genus levels; Table S6. Effects of embryonic synbiotic intervention on the predicted functional KEGG pathways of cecal microbiota in chicks; Table S7. Effects of embryonic synbiotic intervention on plasma biomarkers of intestinal barrier damage in chicks; Figure S1. Hatchability validation in an independent larger-scale experiment. Figure S2. Effects of embryonic SE infection on jejunal and ileal morphology in chicks; Figure S3. Effects of embryonic SE infection on goblet cell numbers in the jejunum and ileum of chicks; Figure S4. Effects of embryonic synbiotic intervention on jejunal and ileal morphology in chicks; Figure S5. Effects of embryonic synbiotic intervention on goblet cell numbers in the jejunum and ileum of chicks.
