# Supplementary material for: Establishment of In Ovo Salmonella Enteritidis Infection and Synbiotic Delivery Models in Chick Embryos and Their Effects on Early Gut Health
Source: Animals (Basel). 2026 Jun 17;16(12):1863. doi: 10.3390/ani16121863 (PMC13295299; doi:10.3390/ani16121863)
Supplement: Supplementary file 1 [file animals-16-01863-s001.zip › Supplementary.pdf]

animals

MDPI

Table S1 Primers used for real-time PCR analyzing

| Genes[1] | | Primers Sequences (5′-3′) | Length (bp) | Accession number |
|---|---|---|---|---|
| PCNA | Forward | GACAATGCGGATACGTTGGC | 188 | NM_204170.2 |
| | Reverse | TCACCAATGTGGCTGAGGTC | | |
| Ki-67 | Forward | CACAGGCAAAGGCTGTCAAA | 164 | XM_015289038.4 |
| | Reverse | TCCGTGCAATTTTCCTTGCT | | |
| Bax | Forward | GTGATGGCATGGGACATAGCTCT | 148 | NM_001025304.1 |
| | Reverse | TGGCGTAGACCTTGCGGATAA | | |
| Caspase3 | Forward | TACCGGACTGTCATCTCGTTCAGG | 162 | XM_015276123.4 |
| | Reverse | ACTGCTTCGCTTGCTGTGATCTTC | | |
| Bcl-2 | Forward | TCGTCGCCTTCTTCGAGTTC | 156 | NM_205339.2 |
| | Reverse | CAAAGGCATCCCATCCTCCG | | |
| Muc2 | Forward | CTTTATAATGTCAGCACCAACTTCTC | 135 | XM_040673077.2 |
| | Reverse | ATTGTGGTAACACCAACATTCATC | | |
| OCLD | Forward | CGTCATGCTCATCGCCTCCATC | 126 | NM_205128.1 |
| | Reverse | TTGAGGTAGGTGCTGCCGTAGG | | |
| CLDN-1 | Forward | GACCAGGTGAAGAAGATGCGGATG | 107 | NM_001013611.2 |
| | Reverse | CGAGCCACTCTGTTGCCATACC | | |
| CLDN-2 | Forward | GGTTCAAGCATCGTGACAGC | 197 | NM_001277622.1 |
| | Reverse | ATGCCCACCACGGCTATAAG | | |
| CLDN-3 | Forward | CCAAGATCACCATCGTCTCC | 113 | NM_204202.2 |
| | Reverse | CACCAGCGGGTTGTAGAAAT | | |
| ZO-1 | Forward | CTTCAGGTGTTTCTCTTCCTCCTC | 131 | XM_015278975.2 |
| | Reverse | CTGTGGTTTCATGGCTGGATC | | |
| ZO-2 | Forward | GCGTCCCATCCTGAGAAATAC | 89 | XM_046934796.1 |
| | Reverse | CTTGTTCACTCCCTTCCTCTTC | | |
| TLR-4 | Forward | TCTTTCAAGGTGCCACATCCA | 152 | KF421483.1 |
| | Reverse | AGCGACGTTAAGCCATGGAA | | |
| MYD88 | Forward | CCGTCTTGTTGGCTCCAGTG | 109 | XM_046921393.1 |
| | Reverse | TGAGGGCAAGAGGACACGAA | | |
| NF-κB | Forward | GTGTGAAGAAACGGGAACTG | 203 | NM_205129.1 |
| | Reverse | GGCACGGTTGTCATAGATGG | | |
| TNF-α | Forward | TGTGTATGTGCAGCAACCCGTAGT | 229 | NM204267 |
| | Reverse | GGCATTGCAATTTGGACAGAAGT | | |
| IL-1β | Forward | GTGAGGCTCAACATTGCGCTGTA | 214 | NM_204524.2 |
| | Reverse | TGTCCAGGCGGTAGAAGATGAAG | | |
| β-actin | Forward | CAGGACTCCATACCCAAGAAAG | 116 | NM_205518.2 |
| | Reverse | TCCCTGGAGAAGAGCTATGAA | | |

[1] PCNA: proliferating cell nuclear antigen; Ki-67: maker of proliferation Ki-67; BAX: BCL-2-associated X protein; Caspase3: cysteine-aspartic acid protease 3; Bcl-2: B-cell lymphoma 2; Muc2: mucin 2; OCLD: occludin; CLDN-1: claudin 1; CLDN-2: claudin 2; CLDN-3: claudin 3; ZO-1: zonula occludens-1; ZO-2: zonula occludens-2; TLR-4: Toll-like receptor 4; MYD88: myeloid differentiation primary response Gene 88; NF-κB: nuclear factor kappa-B; TNF-α: tumor necrosis factor-alpha; IL-1β: interleukin-1 beta.

Table S2 Effects of embryonic SE infection on the relative abundance of cecal microbiota at the phylum and genus levels

| Items | Relative abundance, % | | SEM | *P*-Value |
|---|---|---|---|---|
| | CON | SE1-L | | |
| Actinobacteriota | 4.27 | 0.39 | 1.77 | 0.300 |
| Deinococcota | 0.07 | 0.01 | 0.02 | 0.113 |
| Chloroflexi | 0.04 | 0.01 | 0.01 | 0.332 |
| Myxococcota | 0.04 | 0.03 | 0.01 | 0.785 |
| Verrucomicrobiota | 0.01 | 0.08 | 0.01 | 0.009 |
| Cyanobacteria | 0.00 | 0.51 | 0.11 | 0.009 |
| *Vibrionimonas* | 5.63 | 0.35 | 1.39 | 0.008 |
| *Bradyrhizobium* | 2.11 | 0.18 | 2.11 | 0.008 |
| *Chitinophaga* | 1.91 | 0.00 | 0.45 | 0.008 |
| *Mesorhizobium* | 1.19 | 0.13 | 0.39 | 0.008 |
| *Methylovirgula* | 1.09 | 0.04 | 0.42 | 0.008 |
| *Mycobacterium* | 0.77 | 0.03 | 1.69 | 0.008 |
| *Phyllobacterium* | 0.45 | 0.00 | 0.13 | 0.008 |
| *Asinibacterium* | 0.44 | 0.04 | 0.16 | 0.008 |
| *Rhodanobacter* | 0.18 | 0.01 | 0.04 | 0.008 |
| *Burkholderia-Caballeronia-Paraburkholderia* | 0.15 | 0.0 | 0.38 | 0.032 |
| *Variovorax* | 0.05 | 0.20 | 0.05 | 0.222 |
| *Hydrotalea* | 0.04 | 0.00 | 0.02 | 0.032 |
| *Meiothermus* | 0.03 | 0.00 | 0.02 | 0.056 |
| *Pseudolabrys* | 0.02 | 0.06 | 0.02 | 0.548 |
| *Pajaroellobacter* | 0.01 | 0.03 | 0.01 | 0.548 |
| *Clostridioides* | 0.00 | 0.11 | 0.02 | 0.008 |
| *Epulopiscium* | 0.00 | 0.20 | 0.04 | 0.008 |
| *Paenibacillus* | 0.00 | 0.04 | 0.01 | 0.008 |
| *Terrisporobacter* | 0.00 | 0.04 | 0.01 | 0.015 |
| *Salmonella* | 0.00 | 0.92 | 0.32 | 0.008 |
| *Chloroplast_norank* | 0.00 | 0.20 | 0.06 | 0.032 |
| *Arenimonas* | 0.00 | 0.03 | 0.01 | 0.151 |
| *Methylobacterium-Methylorubrum* | 0.00 | 0.11 | 0.03 | 0.056 |
| *Ralstonia* | 0.00 | 0.04 | 0.02 | 0.056 |
| *PeM15_norank* | 0.00 | 0.13 | 0.03 | 0.008 |

Note: CON: Saline-injected control group; SE1-L: Low-dose *Salmonella* Enteritidis injected group. Data was presented as Means ± SEM, n = 6.

Table S3 Effects of embryonic SE infection on the predicted functional KEGG pathways of cecal microbiota in chicks

| KEGG level | KEGG pathway | Relative abundance, % | | SEM | *P*-Value |
|---|---|---|---|---|---|
| | | CON | SE1-L | | |
| 1 | Metabolism | 68.60 | 66.39 | 0.62 | 0.082 |
| 1 | Genetic Information Processing | 11.25 | 13.03 | 0.30 | 0.054 |
| 1 | Environmental Information Processing | 14.08 | 14.47 | 0.56 | 0.760 |
| 1 | Cellular Processes | 3.43 | 3.27 | 0.13 | 0.561 |
| 1 | Organismal Systems | 0.90 | 0.91 | 0.03 | 0.863 |
| 1 | Human Diseases | 1.70 | 1.93 | 0.12 | 0.096 |
| 2 | Carbohydrate metabolism | 19.09 | 17.65 | 0.35 | 0.038 |
| 2 | Amino acid metabolism | 11.83 | 11.04 | 0.33 | 0.279 |
| 2 | Membrane transport | 9.45 | 10.67 | 0.33 | 0.072 |
| 2 | Energy metabolism | 8.63 | 8.47 | 0.06 | 0.194 |
| 2 | Metabolism of cofactors and vitamins | 6.09 | 7.03 | 0.16 | < 0.001 |
| 2 | Nucleotide metabolism | 5.94 | 6.16 | 0.14 | 0.499 |
| 2 | Signal transduction | 4.63 | 5.28 | 0.26 | 0.261 |
| 2 | Lipid metabolism | 4.29 | 3.78 | 0.16 | 0.133 |
| 2 | Xenobiotics biodegradation and metabolism | 4.27 | 3.17 | 0.41 | 0.220 |
| 2 | Replication and repair | 4.24 | 4.29 | 0.11 | 0.842 |
| 2 | Translation | 4.23 | 4.17 | 0.16 | 0.868 |
| 2 | Metabolism of other amino acids | 3.05 | 3.25 | 0.05 | 0.048 |
| 2 | Folding, sorting and degradation | 2.18 | 2.58 | 0.07 | < 0.001 |
| 2 | Glycan biosynthesis and metabolism | 2.15 | 2.41 | 0.07 | 0.069 |
| 2 | Metabolism of terpenoids and polyketides | 2.12 | 1.84 | 0.09 | 0.146 |
| 2 | Cell motility | 1.97 | 1.71 | 0.13 | 0.391 |
| 2 | Biosynthesis of other secondary metabolites | 1.15 | 1.28 | 0.03 | 0.087 |
| 2 | Cell growth and death | 0.84 | 0.59 | 0.07 | 0.101 |
| 2 | Infectious disease: bacterial | 0.53 | 1.37 | 0.14 | < 0.001 |

Note: CON: Saline-injected control group; SE1-L: Low-dose *Salmonella* Enteritidis injected group. Data was presented as Means ± SEM, n = 6.

Table S4 Effects of embryonic SE infection on plasma biomarkers of intestinal barrier damage in chicks

| Items | Treatments | | SEM | *P*-Value |
|---|---|---|---|---|
| | CON | SE1-L | | |
| LPS (EU/mL) | 0.00 | 3.59 | 0.66 | < 0.001 |
| D-Lactic acid (mmol/mL) | 59.52 | 71.84 | 2.29 | < 0.001 |
| DAO (U/L) | 24.75 | 24.25 | 1.82 | 0.903 |

Note: CON: Saline-injected control group; SE1-L: Low-dose *Salmonella* Enteritidis (strain 1) injected group. Data was presented as Means ± SEM, n = 6.

Table S5 Effects of embryonic synbiotic intervention on the relative abundance of cecal microbiota at the phylum and genus level

| Items | Relative abundance, % | | SEM | *P*-Value |
|---|---|---|---|---|
| | CON | SYN-H | | |
| Actinobacteriota | 4.27 | 0.67 | 1.67 | 0.421 |
| Deinococcota | 0.07 | 0.04 | 0.02 | 0.841 |
| Chloroflexi | 0.02 | 0.04 | 0.01 | 0.421 |
| *Vibrionimonas* | 5.63 | 4.19 | 1.12 | 0.056 |
| *Bradyrhizobium* | 2.11 | 1.48 | 2.04 | 0.056 |
| *Chitinophaga* | 1.91 | 1.01 | 0.38 | 0.056 |
| *Mesorhizobium* | 1.19 | 1.01 | 0.34 | 0.222 |
| *Methylovirgula* | 1.09 | 0.87 | 0.37 | 0.151 |
| *Mycobacterium* | 0.77 | 0.65 | 1.66 | 0.421 |
| *Phyllobacterium* | 0.45 | 0.30 | 0.10 | 0.095 |
| *Asinibacterium* | 0.44 | 0.41 | 0.14 | 0.310 |
| *Rhodanobacter* | 0.18 | 0.05 | 0.03 | 0.016 |
| *Burkholderia-Caballeronia-Para-burkholderia* | 0.15 | 0.06 | 0.38 | 0.008 |
| *Variovorax* | 0.05 | 0.02 | 0.03 | 0.056 |
| *Hydrotalea* | 0.04 | 0.02 | 0.01 | 0.310 |
| *Meiothermus* | 0.03 | 0.03 | 0.02 | 0.841 |
| *Lactiplantibacillus* | 0.00 | 0.06 | 0.02 | 0.151 |

Note: CON: Saline-injected control group; SYN-H: High-dose synbiotic injected group. Data was presented as Means ± SEM, n = 6.

Table S6 Effects of embryonic synbiotic intervention on the predicted functional KEGG pathways of cecal microbiota in chicks

| KEGG level | KEGG pathway | Relative abundance, % | | SEM | *P*-Value |
|---|---|---|---|---|---|
| | | CON | SYN-H | | |
| 1 | Metabolism | 68.60 | 67.07 | 0.54 | 0.189 |
| 1 | Genetic Information Processing | 11.25 | 18.18 | 1.16 | < 0.001 |
| 1 | Environmental Information Processing | 14.08 | 10.37 | 0.76 | 0.009 |
| 1 | Cellular Processes | 3.43 | 2.58 | 0.19 | 0.018 |
| 1 | Organismal Systems | 0.90 | 0.68 | 0.05 | 0.023 |
| 1 | Human Diseases | 1.70 | 1.12 | 0.09 | < 0.001 |
| 2 | Carbohydrate metabolism | 19.09 | 18.97 | 0.27 | 0.849 |
| 2 | Amino acid metabolism | 11.83 | 10.46 | 0.40 | 0.111 |
| 2 | Membrane transport | 9.45 | 7.83 | 0.37 | 0.030 |
| 2 | Energy metabolism | 8.63 | 7.61 | 0.17 | < 0.001 |
| 2 | Metabolism of cofactors and vitamins | 6.09 | 5.59 | 0.13 | 0.050 |
| 2 | Nucleotide metabolism | 5.94 | 8.18 | 0.39 | < 0.001 |
| 2 | Signal transduction | 4.63 | 2.54 | 0.41 | 0.005 |
| 2 | Lipid metabolism | 4.29 | 5.16 | 0.21 | 0.039 |
| 2 | Xenobiotics biodegradation and metabolism | 4.27 | 3.10 | 0.42 | 0.198 |
| 2 | Replication and repair | 4.24 | 6.76 | 0.42 | < 0.001 |
| 2 | Translation | 4.23 | 7.59 | 0.57 | < 0.001 |
| 2 | Metabolism of other amino acids | 3.05 | 2.88 | 0.05 | 0.127 |
| 2 | Folding, sorting and degradation | 2.18 | 2.86 | 0.11 | < 0.001 |
| 2 | Glycan biosynthesis and metabolism | 2.15 | 1.79 | 0.08 | 0.022 |
| 2 | Metabolism of terpenoids and polyketides | 2.12 | 2.30 | 0.08 | 0.318 |
| 2 | Cell motility | 1.97 | 1.00 | 0.22 | 0.022 |
| 2 | Biosynthesis of other secondary metabolites | 1.15 | 1.03 | 0.05 | 0.281 |
| 2 | Cell growth and death | 0.84 | 1.13 | 0.08 | 0.060 |
| 2 | Infectious disease: bacterial | 0.53 | 0.36 | 0.05 | 0.139 |

Note: CON: Saline-injected control group; SYN-H: High-dose synbiotic injected group. Data was presented as Means ± SEM, n = 6.

Table S7 Effects of embryonic synbiotic intervention on plasma biomarkers of intestinal barrier damage in chicks

| Items | Treatments | | SEM | *P*-Value |
|---|---|---|---|---|
| | CON | SYN-H | | |
| LPS (EU/mL) | 0.00 | 0.00 | — | — |
| D-Lactic acid (mmol/mL) | 59.52 | 57.91 | 0.63 | 0.215 |
| DAO (U/L) | 24.75 | 19.00 | 2.07 | 0.180 |

**Note:** CON: saline-injected control group; SYN-H: high-dose synbiotic group. Data were presented as Means ± SEM, n = 6.

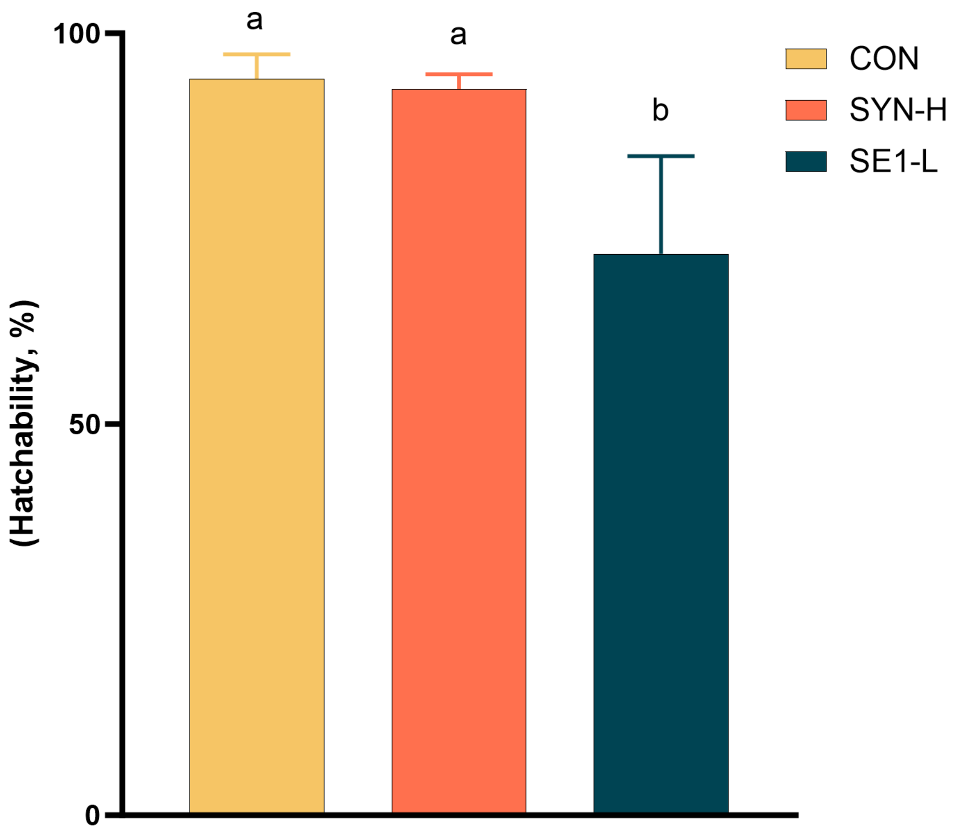


**Figure S1. Hatchability validation in an independent larger-scale experiment.** Hatchability of chicks in the CON, SYN-H, and SE1-L groups was evaluated in an independent larger-scale validation experiment conducted under the same incubation and in ovo injection procedures as the initial model-establishment experiment. A total of 750 eggs were initially incubated. After removal of infertile eggs and eggs containing dead embryos, 720 fertilized eggs were allocated to three treatment groups, with 240 eggs per group. Each group contained six replicate trays, with 40 fertilized eggs per replicate tray. Hatchability was calculated for each replicate tray. Data are presented as means ± SEM (n = 6 replicate trays per group). Different lowercase letters indicate significant differences among groups ($p < 0.05$).

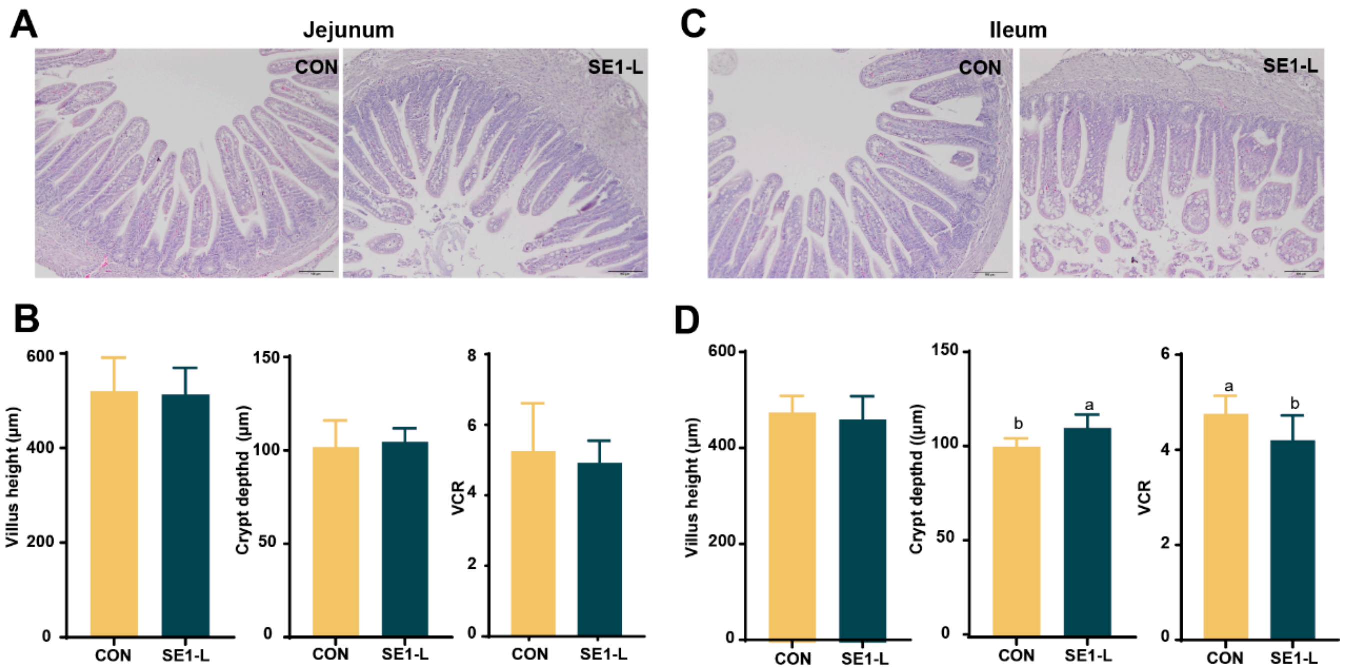


**Figure S2. Effects of embryonic SE infection on jejunal and ileal morphology in chicks.** (A) Jejunal H&E staining images (100×); (B) jejunal morphometric parameters; (C) ileal H&E staining images (100×); (D) ileal morphometric parameters. VCR, villus height-to-crypt depth ratio. CON, saline-injected control group; SE1-L, low-dose *Salmonella* Enteritidis strain 1 group. Data are presented as means ± SEM (n = 6). Different lowercase letters indicate significant differences between groups ($p < 0.05$).

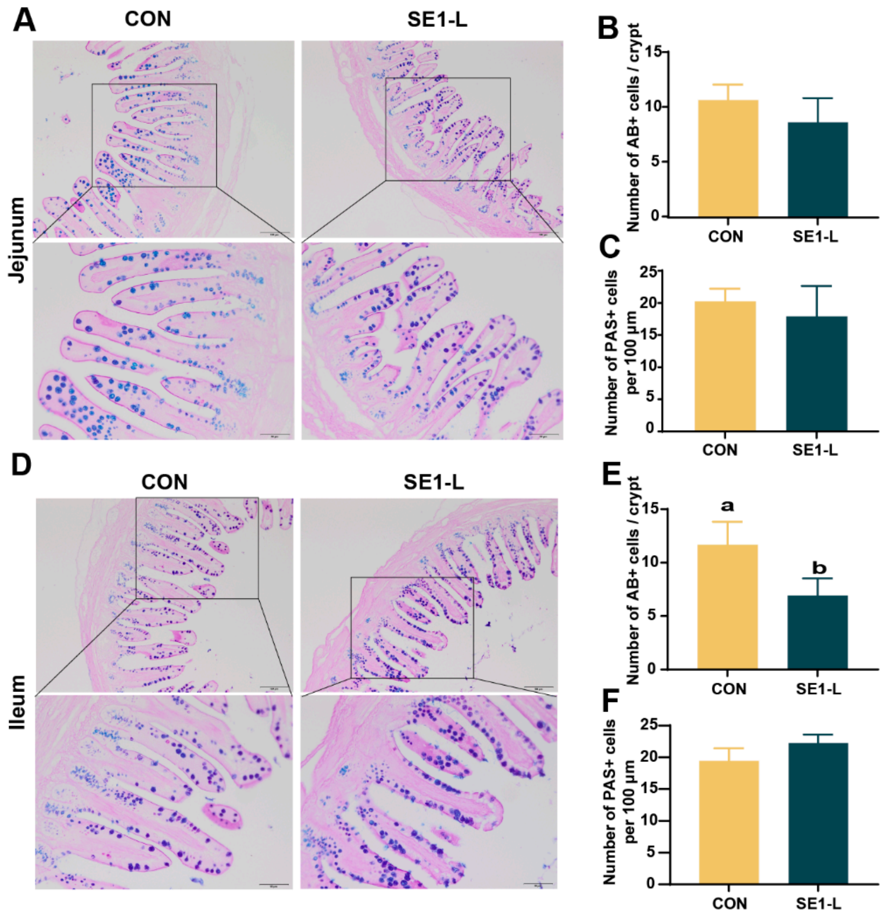


**Figure S3. Effects of embryonic SE infection on goblet cell numbers in the jejunum and ileum of chicks.** (A) Jejunal AB-PAS staining images; (B) quantification of AB-positive goblet cells within jejunal crypts; (C) quantification of PAS-positive goblet cells per 100 μm of jejunal villus length; (D) ileal AB-PAS staining images; (E) quantification of AB-positive goblet cells within ileal crypts; (F) quantification of PAS-positive goblet cells per 100 μm of ileal villus length. CON, saline-injected control group; SE1-L, low-dose *Salmonella* Enteritidis strain 1 group. Data are presented as means ± SEM (n = 6). Different lowercase letters indicate significant differences between groups ($p < 0.05$).

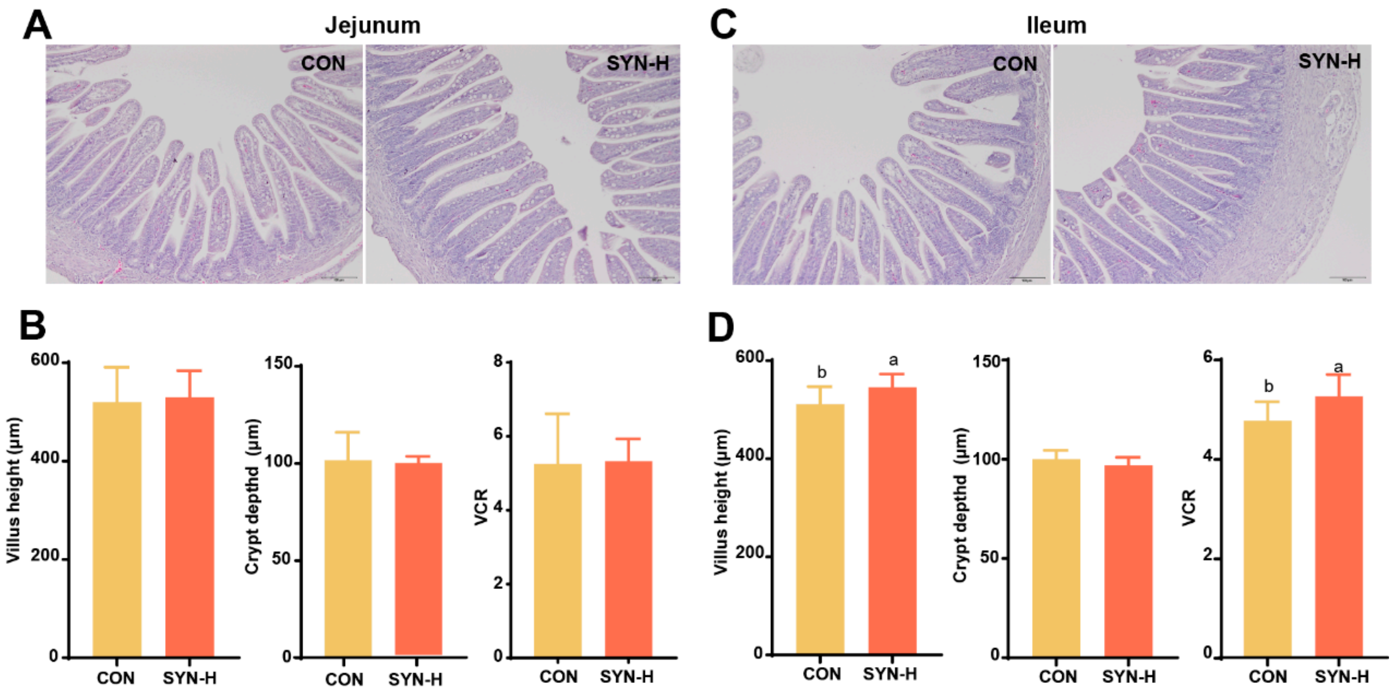


**Figure S4. Effects of embryonic synbiotic intervention on jejunal and ileal morphology in chicks.** (A) Jejunal H&E staining images (100×); (B) jejunal morphometric parameters; (C) ileal H&E staining images (100×); (D) ileal morphometric parameters. VCR, villus height-to-crypt depth ratio. CON, saline-injected control group; SYN-H, high-dose synbiotic group. Data are presented as means ± SEM (n = 6). Different lowercase letters indicate significant differences between groups ($p < 0.05$).

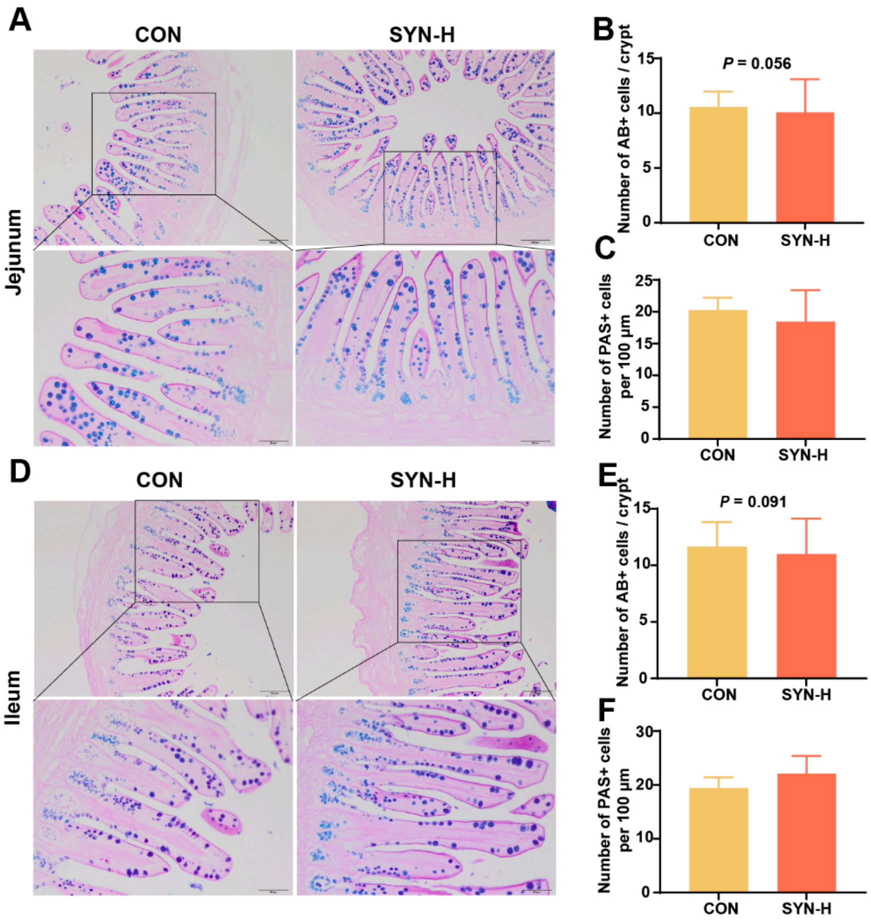


**Figure S5. Effects of embryonic synbiotic intervention on goblet cell numbers in the jejunum and ileum of chicks.** (A) Jejunal AB-PAS staining images; (B) quantification of AB-positive goblet cells within jejunal crypts; (C) quantification of PAS-positive goblet cells per 100 μm of jejunal villus length; (D) ileal AB-PAS staining images; (E) quantification of AB-positive goblet cells within ileal crypts; (F) quantification of PAS-positive goblet cells per 100 μm of ileal villus length. CON, saline-injected control group; SYN-H, high-dose synbiotic group. Data are presented as means ± SEM (n = 6). Different lowercase letters indicate significant differences between groups ($p < 0.05$).
