# Supplementary figures and images for: Establishment of In Ovo Salmonella Enteritidis Infection and Synbiotic Delivery Models in Chick Embryos and Their Effects on Early Gut Health
Source: Animals (Basel). 2026 Jun 17;16(12):1863. doi: 10.3390/ani16121863 (PMC13295299; doi:10.3390/ani16121863)

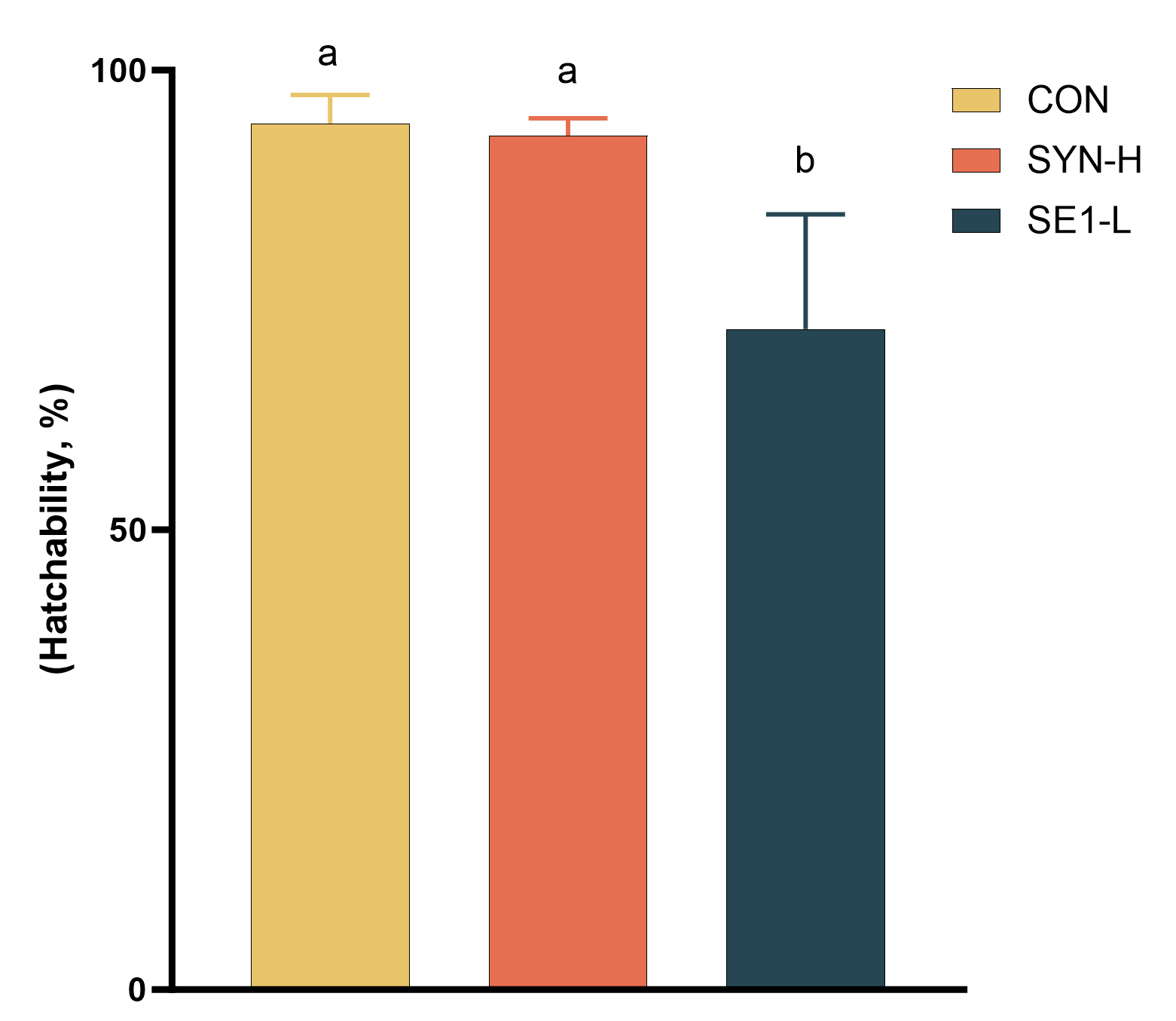

Supplement: Supplementary file 1 [file animals-16-01863-s001.zip › Fig.S1.tif]

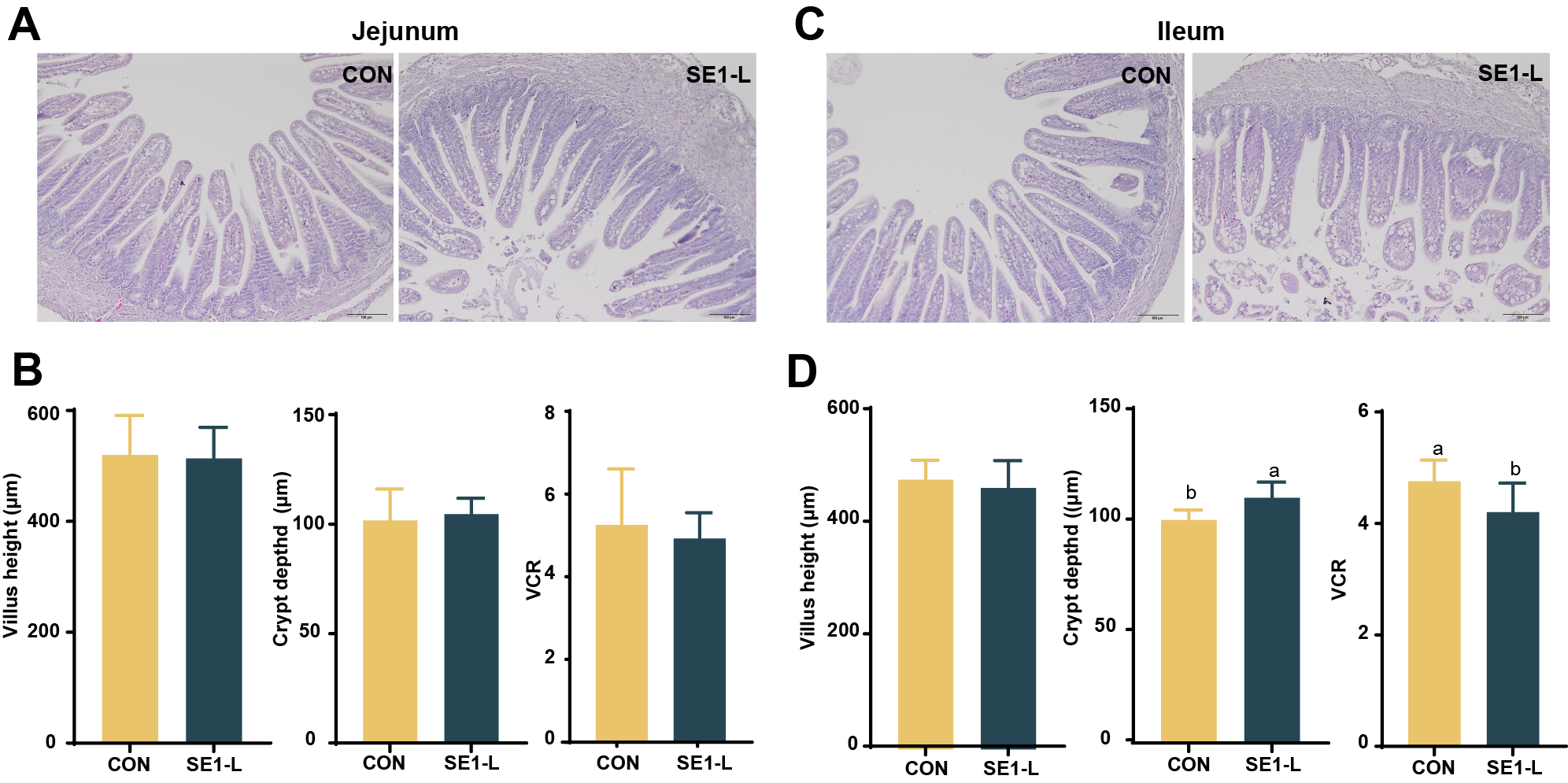

Supplement: Supplementary file 1 [file animals-16-01863-s001.zip › Fig.S2.tif]

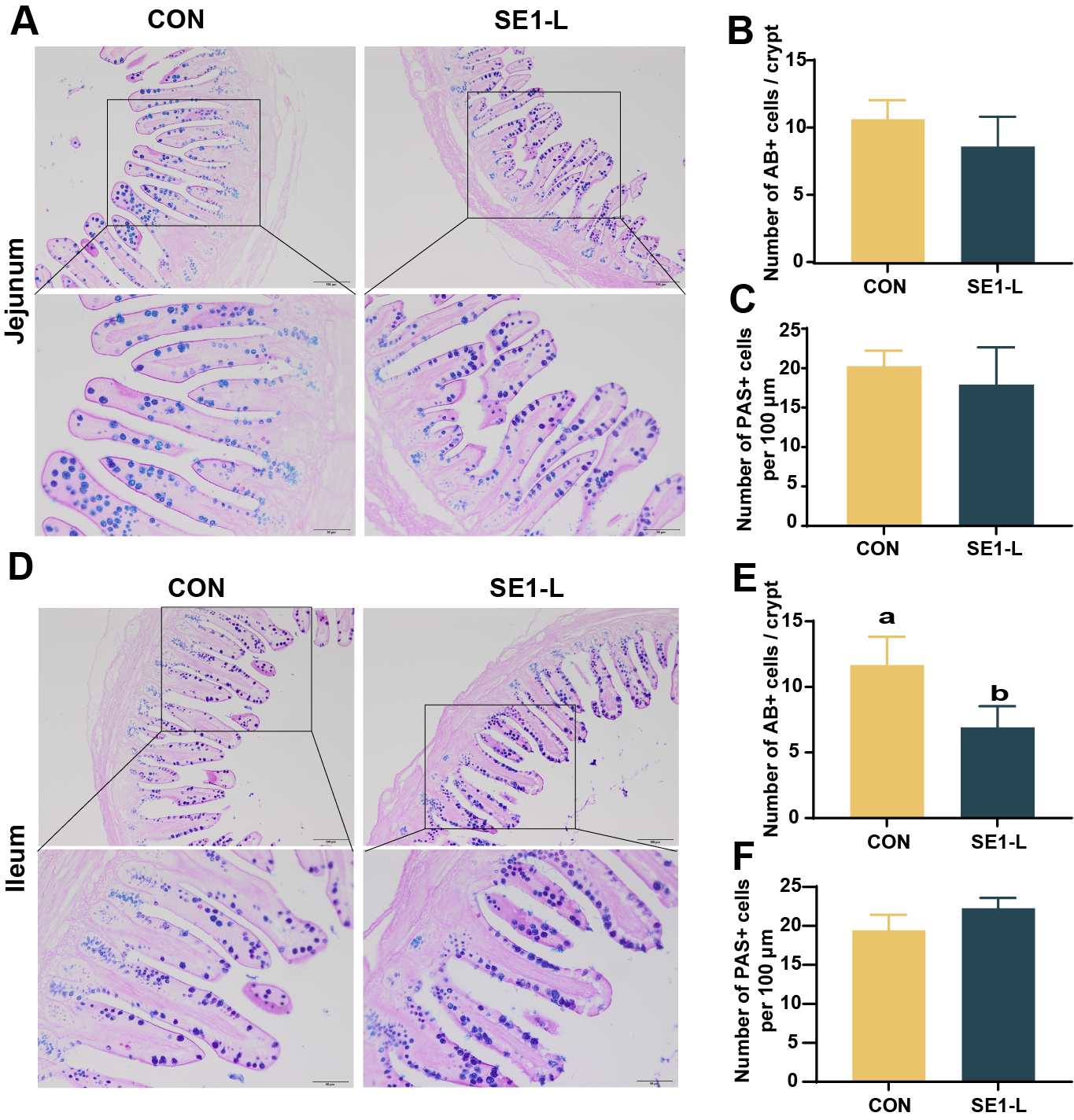

Supplement: Supplementary file 1 [file animals-16-01863-s001.zip › Fig.S3.tif]

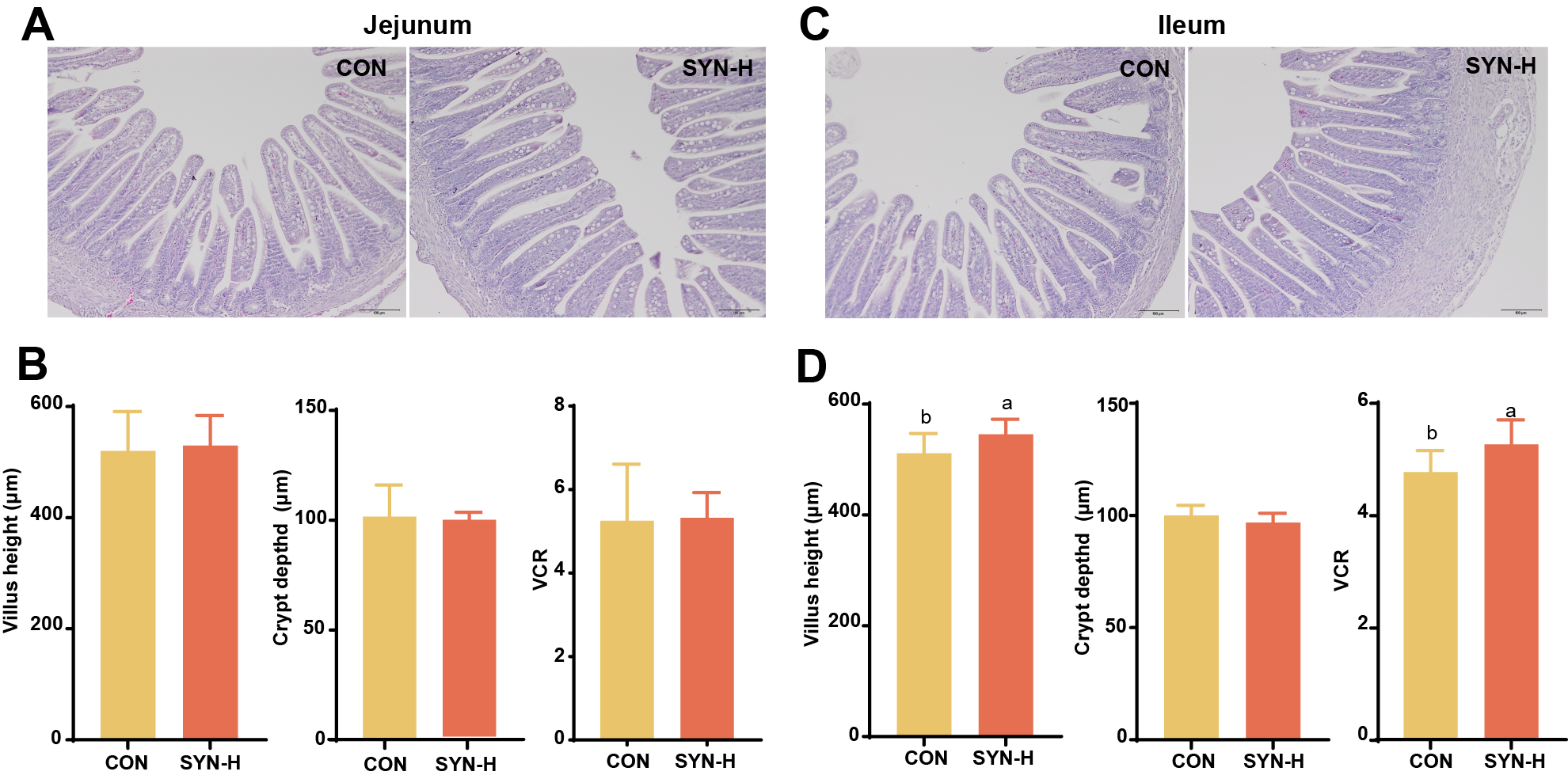

Supplement: Supplementary file 1 [file animals-16-01863-s001.zip › Fig.S4.tif]

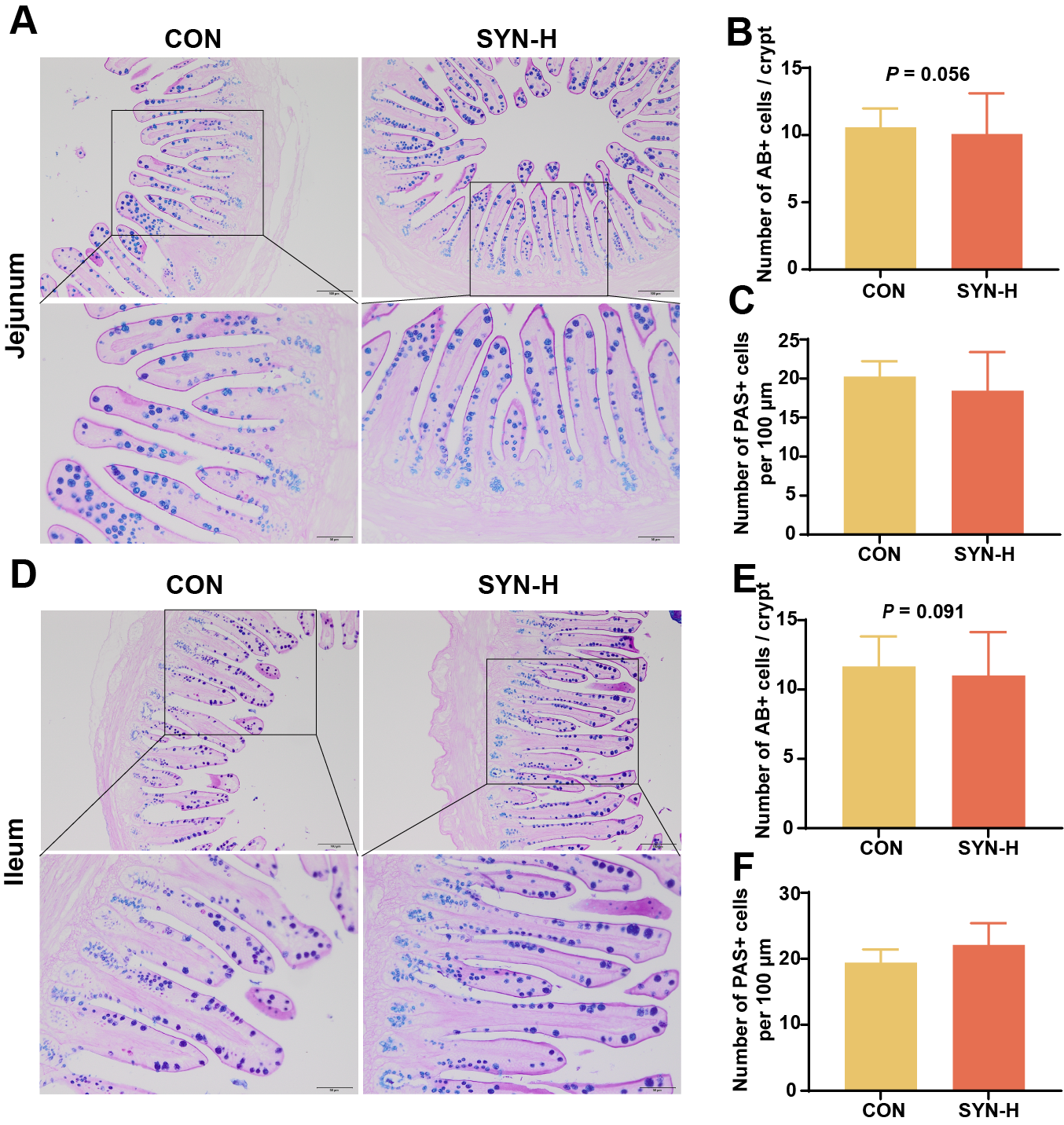

Supplement: Supplementary file 1 [file animals-16-01863-s001.zip › Fig.S5.tif]
